# Classifying pairs with trees for supervised biological network inference†
†Electronic supplementary information (ESI) available: Implementation and computational issues, supplementary performance curves, and illustration of interpretability of trees. See DOI: 10.1039/c5mb00174a



**DOI:** 10.1039/c5mb00174a

**Published:** 2015-05-26

**Authors:** Marie Schrynemackers, Louis Wehenkel, M. Madan Babu, Pierre Geurts

**Affiliations:** a Department of EE and CS & GIGA-R , University of Liège , Belgium . Email: marie.schrynemackers@ulg.ac.be; b MRC Laboratory of Molecular Biology , Cambridge , UK

## Abstract

Networks are ubiquitous in biology, and computational approaches have been largely investigated for their inference. In particular, supervised machine learning methods can be used to complete a partially known network by integrating various measurements. Two main supervised frameworks have been proposed: the local approach, which trains a separate model for each network node, and the global approach, which trains a single model over pairs of nodes. Here, we systematically investigate, theoretically and empirically, the exploitation of tree-based ensemble methods in the context of these two approaches for biological network inference. We first formalize the problem of network inference as a classification of pairs, unifying in the process homogeneous and bipartite graphs and discussing two main sampling schemes. We then present the global and the local approaches, extending the latter for the prediction of interactions between two unseen network nodes, and discuss their specializations to tree-based ensemble methods, highlighting their interpretability and drawing links with clustering techniques. Extensive computational experiments are carried out with these methods on various biological networks that clearly highlight that these methods are competitive with existing methods.

## Abstract 2

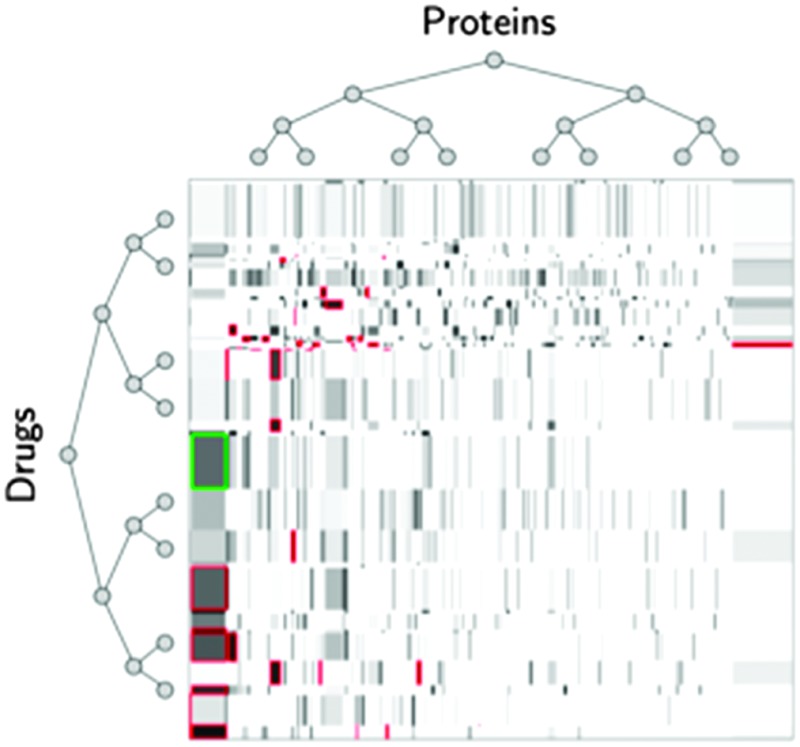
We systematically investigate, theoretically and empirically, the application of tree-based methods for the supervised inference of biological networks.

## 1 Introduction

In biology, the relationship between biological entities (genes, proteins, transcription factors, micro-RNA, diseases, *etc.*) is often represented by graphs (or networks‡
‡In this paper, the terms network and graph will refer to the same thing.). In theory, most of these networks can be identified from lab experiments but in practice, because of the difficulties in setting up these experiments and their costs, we often have only a very partial knowledge of them. Because more and more experimental data become available about biological entities of interest, several researchers took an interest in using computational approaches to predict interactions between nodes in order to complete experimental predictions.

When formulated as a supervised learning problem, network inference consists in learning a classifier on pairs of nodes. Mainly two approaches have been investigated in the literature to adapt existing classification methods for this problem.^1^ The first one, that we call the global approach, considers this problem as a standard classification problem on an input feature vector obtained by concatenating the feature vectors of each node from the pair.^1^ The second approach, called local,^2,3^ trains a different classifier for each node separately, aiming at predicting its direct neighbors in the graph. These two approaches have been mainly exploited with support vector machine (SVM) classifiers. In particular, several kernels have been proposed for comparing pairs of nodes in the global approach^4,5^ and the global and local approaches can be related for specific choices of this kernel.^6^ A number of papers applied the global approach with tree-based ensemble methods, mainly Random Forests,^7^ for the prediction of protein–protein^8–11^ and drug–protein^12^ interactions, combining various feature sets. Besides the local and global methods, other approaches for the supervised graph inference includes, among others, matrix completion methods,^13^ methods based on output kernel regression,^14,15^ Random Forests-based similarity learning,^16^ and methods based on network properties.^17^

In this paper, we would like to systematically investigate, theoretically and empirically, the exploitation of tree-based ensemble methods in the context of the local and global approaches for supervised biological network inference. We first formalize biological network inference as the problem of classification of pairs, considering in the same framework homogeneous graphs, defined on one kind of nodes, and bipartite graphs, linking nodes of two families. We then define the general local and global approaches in the context of this formalization, extending in the process the local approach for the prediction of interactions between two unseen network nodes. The paper discusses in details the specialization of these approaches to tree-based ensemble methods. In particular, we highlight their high potential in terms of interpretability and draw connections between these methods and unsupervised (bi-)clustering methods. Experiments on several biological networks show the good predictive performance of the resulting family of methods. Both the local and the global approaches are competitive with however an advantage for the global approach in terms of predictive performance and for the local approach in terms of compactness of the inferred models.

The paper is structured as follows. Section 2 first defines the general problem of supervised network inference and cast it as a classification problem on pairs. Then, it presents two generic approaches to address it and their particularization for tree ensembles. Section 3 reports experiments with these methods on several homogeneous and bipartite biological networks. Section 4 concludes and discusses future work directions. Additional experimental results and implementation details can be found in the ESI.†

## 2 Methods

We first formalize the problem of supervised network inference and discuss the evaluation of these methods in Section 2.1. We then present in Section 2.2 two generic approaches to address it. Section 2.3 discusses the specialization of these two approaches in the context of tree-based ensemble methods.

### 2.1 Supervised network inference as classification on pairs

**Fig. 1:**
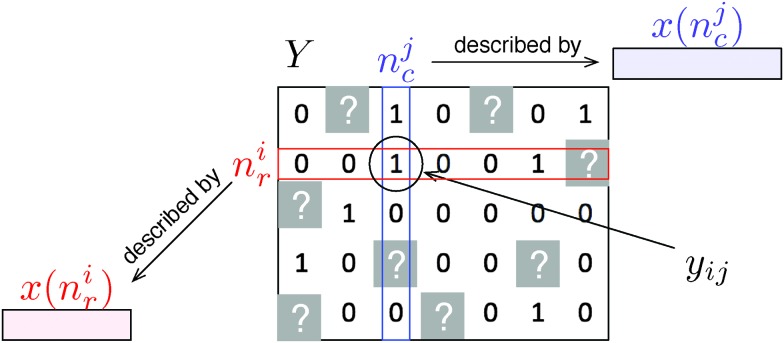
A network can be represented by an adjacency matrix *Y* where each row and each column correspond to a specific node, with potentially different families of nodes associated with rows and columns. Each node is furthermore described by a feature vector, with potentially different features describing row and column nodes. For instance, row nodes n*i*r can be proteins and column nodes n*j*c can be drugs, with the adjacency matrix encoding drug–protein interactions. Proteins could be described by their PFAM domains and drugs by features encoding their chemical structure. Supervised network inference then consists of inferring missing entries in the adjacency matrix (question marks in gray) from known entries (in white) by exploiting node features.

For the sake of generality, we consider bipartite graphs that connect two sets of nodes. The graph is thus defined by an adjacency matrix *Y*, where each entry *y*_*ij*_ is equal to one if there is an edge between the nodes n*i*r and n*j*c, and zero if not. The subscripts r and c are used to differentiate the two sets of nodes and stand, respectively, for *row* and *column* of the adjacency matrix *Y*. Moreover, each node (or sometimes pair of nodes) is described by a feature representation, *i.e.* typically a vector of numerical values, denoted by *x*(n) (see Fig. 1 for an illustration). Homogeneous graphs defined for only one family of nodes can be obtained as special cases of this general framework by considering only one set of nodes and thus a square and symmetric adjacency matrix.^18^

In this context, the problem of supervised network inference can be formulated as follows (see Fig. 1):

Given a partial knowledge of the adjacency matrix *Y* of the target network, find the best possible predictions of the missing or unknown entries of this matrix by exploiting the feature description of the network nodes.

In this paper, we address this problem as a supervised classification problem on pairs.^18^ A learning sample, denoted LS_p_, is constructed as the set of all pairs of nodes that are known to interact or not (*i.e.*, the known entries in the adjacency matrix). The input variables used to describe these pairs are the feature vectors of the two nodes in the pair. A classification model f (*i.e.* a function associating a label in {0,1} to each combination of the input variables) can then be trained from LS_p_ and used to predict the missing entries of the adjacency matrix.

The evaluation of the predictions of the supervised network inference methods requires special care. Indeed, all pairs are not as easy as the others to predict: it is typically much more difficult to predict pairs that involve nodes for which no examples of interactions are provided in the learning sample LS_p_. As a consequence, to get a complete assessment of a given method, one needs to partition the predictions into different families, depending on whether the nodes in the tested pair are represented or not in the learning set LS_p_, and then to perform a separate evaluation within each family.^18^

To formalize this, let us denote by LS_c_ and LS_r_ the nodes from the two sets that are present in LS_p_ (*i.e.* which are involved in some pairs in LS_p_) and by TS_c_ and TS_r_ (where TS stands for the test set) the nodes that are unseen in LS_p_. The pairs of nodes to predict (*i.e.*, outside LS_p_) can be divided into the following four families (where *S*_1_ × *S*_2_ denotes the cartesian product between sets *S*_1_ and *S*_2_ and *S*_1_/*S*_2_ their difference):

• (LS_r_ × LS_c_)/LS_p_: predictions of (unseen) pairs between two nodes which are represented in the learning sample.

• LS_r_ × TS_c_ or TS_r_ × LS_c_: predictions of pairs between one node represented in the learning sample and one unseen node.

• TS_r_ × TS_c_: predictions of pairs between two unseen nodes.

**Fig. 2:**
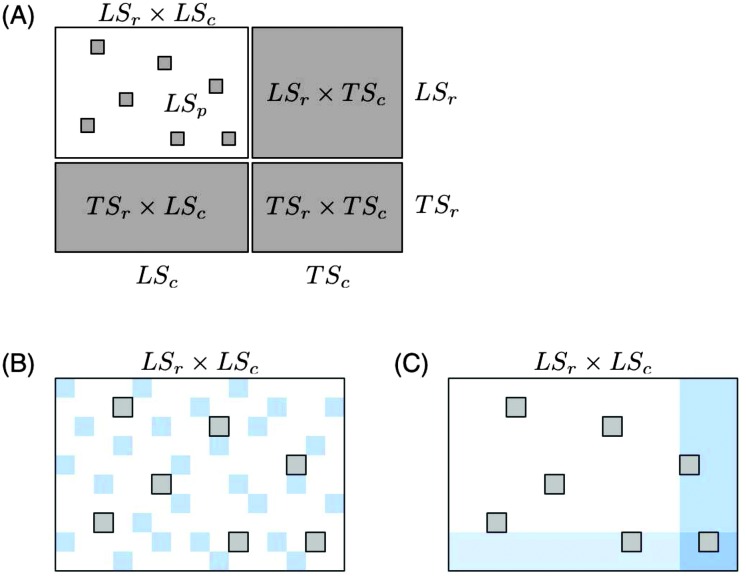
(A) Schematic representation of known and unknown pairs in the network adjacency matrix. Known pairs (that can be interacting or not) are in white and unknown pairs, to be predicted, are in gray. Rows and columns of the adjacency matrix have been rearranged to highlight the four families of unknown pairs described in the text: LS_r_ × LS_c_, LS_r_ × TS_c_, TS_r_ × LS_c_, and TS_r_ × TS_c_. (B) Schematic representation of CV on pairs: in this procedure, we randomly divide the pairs of the learning sample into two groups: we learn a model on the pairs from the white area, and test it on the pairs from the blue area. The CV on pairs evaluates LS × LS predictions. Pairs in gray represent unknown pairs that do not take part to the CV. (C) Schematic representation of CV on nodes: in this procedure, we randomly divide the nodes of each set (relative to the rows and the columns) into two groups: we learn a model on the pairs from the white area, and test it on the pairs from the blue area. The CV on pairs evaluates LS × TS, TS × LS and TS × TS predictions.

These families of pairs are represented in the adjacency matrix in Fig. 2A. Thereafter, to simplify the notations, we denote the four families as LS × LS, LS × TS, TS × LS and TS × TS. In the case of an homogeneous undirected graph, only three sets can be defined as the two sets LS × TS and TS × LS are confounded.^18^

Prediction performances are expected to differ between these four families. Typically, one expects that TS × TS pairs will be the most difficult to predict since less information is available at training about the corresponding nodes. These predictions will then be evaluated separately in this work, as suggested in several publications.^18,19^ They can be evaluated by performing two kinds of cross-validation (CV): a first CV procedure on pairs of nodes (denoted “CV on pairs”) to evaluate LS × LS predictions (see Fig. 2B) and a second CV procedure on nodes (denoted “CV on nodes”) to evaluate LS × TS, TS × LS and TS × TS predictions (see Fig. 2C).^18^

### 2.2 Two different approaches

In this section, we present the two generic, local and global, approaches we have adopted for dealing with classification on pairs. We will discuss in Section 2.3 their practical implementation in the context of tree-based ensemble methods. In the presentation of the approaches, we will assume that we have at our disposal a classification method that derives its classification model from a class conditional probability model. Denoting by *f* a classification model, we will denote by *f* ^p^ (*i.e.*, with superscript p) the corresponding class conditional probability function. *f*(*x*) is the predicted class (0 or 1) associated with some input *x*, while *f* ^p^(*x*) (resp. 1 – *f* ^p^(*x*)) is the predicted probability (∈[0,1]) of the input *x* being of class 1 (resp. 0). Typically, *f*(*x*) is obtained from *f* ^p^(*x*) by computing *f*(*x*) = 1(*f* ^p^(*x*) > *p*_th_) for some user-defined threshold *p*_th_ ∈ [0,1], where *p*_th_ can be adjusted to find the best tradeoff between sensitivity and specificity according to the application needs.

#### 2.2.1 Global approach

**Fig. 3:**
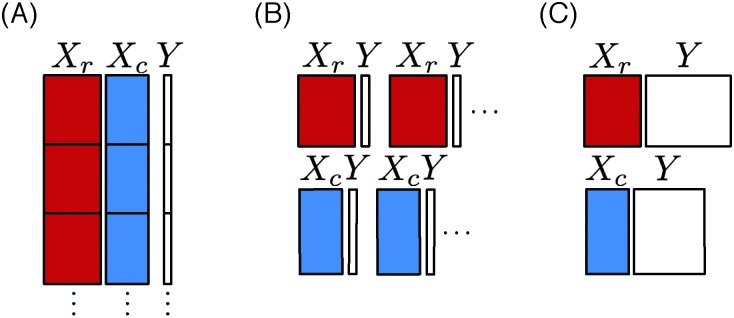
Schematic representation of the training data. In the global approach (A) the features vectors are concatenated, in the local approach with single output (B) one function is learnt for each node, and in the local approach with multiple output (C) one function is learnt for one family of nodes and one function for the other one.

The most straightforward approach for dealing with the problem defined in Section 2.1 is to apply a classification algorithm on the learning sample LS_p_ of pairs to learn a function *f*_glob_ on the cartesian product of the two input spaces (resulting in the concatenation of the two input vectors of the nodes of the pair). Predictions can then be computed straightforwardly for any new unseen pair from the function (Fig. 3A).

In the case of a homogeneous graph, the adjacency matrix *Y* is a symmetric square matrix. We will introduce two adaptations of the approach to handle such graphs. First, for each pair (n_r_,n_c_) in the learning sample, the pair (n_c_,n_r_) will also be introduced in the learning sample. Without further constraint on the classification method, this will not ensure however that the learnt function *f*_glob_ will be symmetric in its arguments. To make it symmetric, we will compute a new class conditional probability model *f*pglob,sym from the learned one *f*pglob as follows:
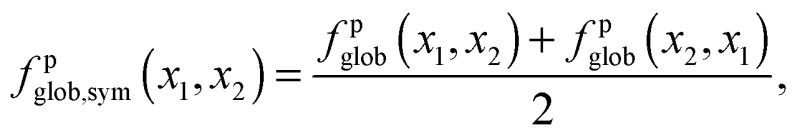
where *x*_1_ and *x*_2_ are the input feature vectors of the nodes in the pair to be predicted.

#### 2.2.2 Local approach

The idea of the local approach,^2^ is to build a separate classification model for each node, trying to predict its neighbors in the graph from the known graph around this node. More precisely, for a given node n_c_ ∈ LS_c_, a new learning sample LS(n_c_) is constructed from the learning sample of pairs LS_p_, comprising all the pairs that involve the target node n_c_ and the feature vectors associated with the interacting nodes n_r_. It can then be used to learn a classification model *f*_n_c__, which can be exploited to make a prediction for any new pair involving n_c_. By symmetry, the same strategy can be adopted to learn a classification model *f*_n_r__ for each node n_r_ ∈ LS_r_ (Fig. 3B).

These two sets of classifiers can then be exploited to make LS × TS and TS × LS types of predictions. For pairs (n_r_,n_c_) in LS × LS, two predictions can be obtained: *f*_n_c__(n_r_) and *f*_n_r__(n_c_). We propose to simply combine them by an arithmetic average of the corresponding class conditional probability estimates.

**Fig. 4:**
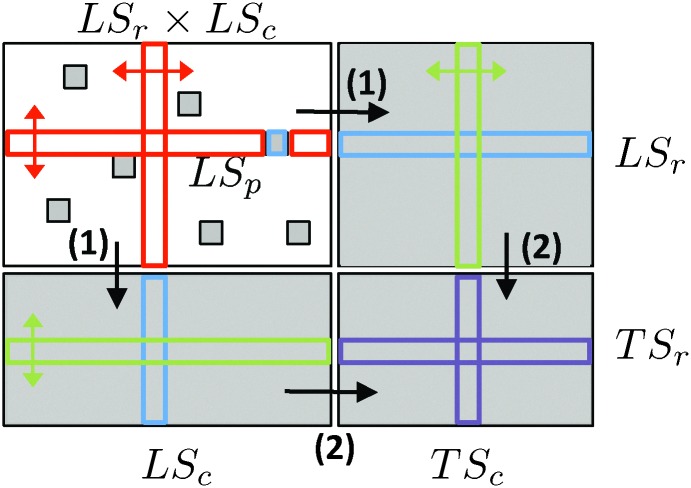
The local approach needs two steps to learn a classifier for an unseen node: (1) first, we predict LS × TS and TS × LS interactions, and (2) from these predictions, we predict TS × TS interactions.

As such, the local approach is in principle not able to make direct predictions for pairs of nodes (n_r_,n_c_) ∈ TS × TS (because LS(n_r_) = LS(n_c_) = ⊘ for n_r_ ∈ TS_r_ and n_c_ ∈ TS_c_). We nevertheless propose to use the following two-step procedure to learn a classifier for a node n_r_ ∈ TS_r_ (see Fig. 4):

• First, learn all classifiers *f*_n_c__ for nodes n_c_ ∈ LS_c_ (equivalent to the completion of the columns in Fig. 4),

• Then, learn a classifier 
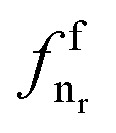
 from the predictions given by the models *f*_n_c__ trained in the first step (equivalent to the completion of the rows in Fig. 4).

Again by symmetry, the same strategy can be applied to obtain models 
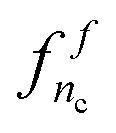
 for the nodes n_c_ ∈ TS_c_. A prediction is then obtained for a pair (n_r_,n_c_) in TS × TS by averaging the class conditional probability predictions of both models 
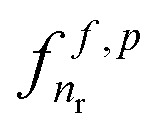
 and 
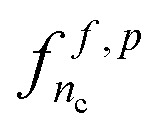
. A related two-step procedure has been proposed by Pahikkala *et al.* ^20^ for learning on pairs with kernel methods.

Note that to derive the learning samples needed to train models 
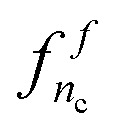
 and 
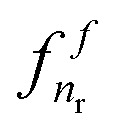
 in the second step, one requires to choose a threshold on the predicted class conditional probability estimates (to turn these probabilities into binary classes). In our experiments, we will set this threshold in such a way that the proportion of edges *versus* non edges in the predicted subnetworks in LS × TS and TS × LS is equal to the same proportion within the original learning sample of pairs.

This strategy can be specialized to the case of a homogeneous graph in a straightforward way. Only one class of classifiers *f*_n_ and *f*fn are trained for nodes in LS and in TS respectively (using the same two-step procedure as in the asymmetric case for the second). LS × LS and TS × TS predictions are still obtained by averaging two predictions, one for each node of the pair.

### 2.3 Tree-based ensemble methods

Any method could be used as a base classifier for the two approaches. In this paper, we propose to evaluate the use of tree-based ensemble methods in this context. We first briefly describe these methods and then discuss several aspects related to their use within the two generic approaches.

#### 2.3.1 Description of the methods

A decision tree^21^ represents an input–output model by a tree whose interior nodes are each labeled with a (typically binary) test based on one input feature and each terminal node is labeled with a value of the output. The predicted output for a new instance is determined as the output associated with the leaf reached by the instance when it is propagated through the tree starting at the root node. A tree is built from a learning sample of input–output pairs, by recursively identifying, at each node, the test that leads to a split of the node sample into two subsamples that are as pure as possible in terms of their output values.

Single decision trees typically suffer from high variance, which makes them not competitive in terms of accuracy. This problem is circumvented by using ensemble methods that generate several trees and then aggregate their predictions. In this paper, we exploit one particular ensemble method called extremely randomized trees (extra-trees^22^). This method grows each tree in the ensemble by selecting at each node the best among *K* randomly generated splits. In our experiments, we use the default setting of *K*, equal to the square root of the total number of candidate attributes.

One interesting feature of tree-based methods (single and ensemble) is that they can be extended to predict a vectorial output instead of a single scalar output.^23^ We will exploit this feature of the method in the context of the local approach below.

#### 2.3.2 Global approach

**Fig. 5:**
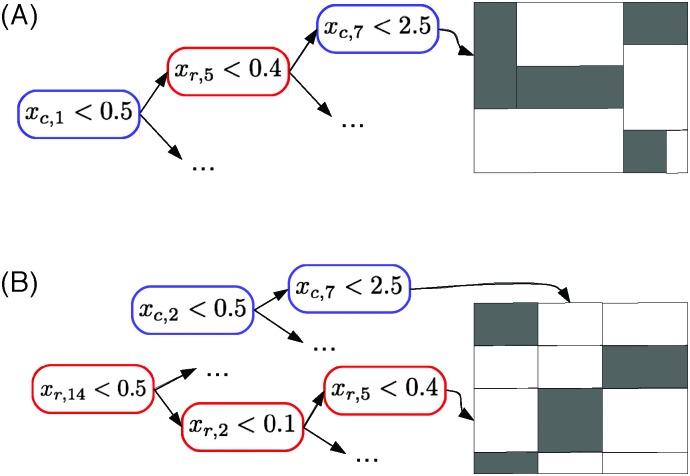
Both the global approach (A) and the local approach with multiple output (B) can be interpreted as carrying out a biclustering of the adjacency matrix. Each subregion is characterized by conjunctions of tests based on the input features. In this graph, *x*_c,*i*_ (resp. *x*_r,*i*_) denotes the *i*th feature of the column (resp. row) node. Note that in the case of the global approach, the representation is only illustrative. The adjacency submatrices corresponding to the tree leaves can not be necessarily rearranged as contiguous rectangular submatrices covering the initial adjacency matrix.

The global approach consists of building a tree from the learning sample of all pairs. Each split of the resulting tree will be based on one of the input features coming from either one of the two input feature vectors, *x*(n_r_) or *x*(n_c_). The tree growing procedure can thus be interpreted as interleaving the construction of two trees: one on the row nodes and one on the column nodes. Each leaf of the resulting tree is thus associated with a rectangular submatrix of the graph adjacency matrix *Y* (reduced to the pairs in LS_r_ × LS_c_) and the construction of the tree is such that the pairs in this submatrix should be, as far as possible, either all connected or all disconnected (see Fig. 5 for an illustration).

#### 2.3.3 Local approach

The use of tree ensembles in the context of the local approach is straightforward. We will nevertheless compare two variants. The first one builds a separate model for each row and column nodes as presented in Section 2.2. The second method exploits the ability of tree-based methods to deal with multiple outputs (vector outputs) to build only two models, one for the row nodes and one for the column nodes (Fig. 3C). We assume that the learning sample has been generated by sampling two subsets of nodes LS_r_ and LS_c_ and that the full adjacency matrix is observed between these two sets (as in Fig. 2C). The first model related to the column nodes is built from a learning sample LS(n_c_) comprising all the observed pairs, and the feature vectors associated with the row nodes n_r_. It can then be used to learn a classification model, which can be exploited to make the predictions of the interaction profiles of all nodes n_c_ present in the learning sample of LS_p_ pairs. By symmetry, the same strategy can be adopted to learn classification model for the row nodes n_r_. The two-step procedure can then be applied to build the two models required to make TS × TS predictions.

This approach has the advantage of requiring only four tree ensemble models in total instead of one model for each potential node in the case of the single output approach. It can however only be used when the complete submatrix is observed for pairs in LS × LS, since the tree-based ensemble method cannot cope with missing output values.

#### 2.3.4 Interpretability

One main advantage of tree-based methods is their interpretability, directly through the tree structure in the case of single tree models and through feature importance rankings in the case of ensembles.^24^ Let us compare both approaches along this criterion.

In the case of the global approach, as illustrated in Fig. 5A, the tree that is built partitions the adjacency matrix (more precisely, its LS_r_ × LS_c_ part) into rectangular regions. These regions are defined such that pairs in each region are either all connected or all disconnected. The region is furthermore characterized by a path in the tree (from the root to the leaf) corresponding to tests on the input features of both nodes of the pair.

**Fig. 6:**
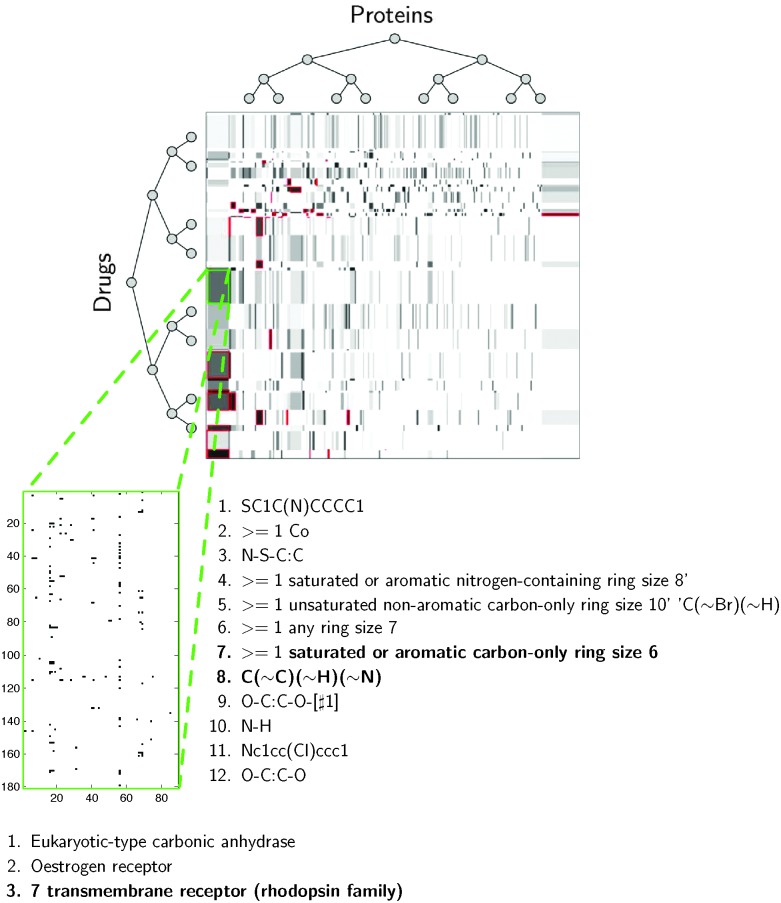
Illustration of the interpretability of multiple-output decision-tree on a drug–protein interaction network. We zoomed in the rectangular subregion with the highest number of interactions, and presented a list of drug and protein features associated with this region. See the ESI† for more details about the procedures.

In the case of the local multiple output approach, one of the two trees partitions the rows and the other tree partitions the columns of the adjacency matrix. Each partitioning is carried out in such a way that nodes in each subpartition have a similar connectivity profile. The resulting partitioning of the adjacency matrix will thus follow a checkerboard structure with also only connected or disconnected pairs in the obtained submatrix, as far as possible (Fig. 5B). Each submatrix will be furthermore characterized by two conjunctions of tests, one based on row inputs and one based on column inputs. These two methods can thus be interpreted as carrying out a biclustering^25^ of the adjacency matrix where the biclustering is however directed by the choice of tests on the input features. A concrete illustration can be found in Fig. 6 and in the ESI.†

In the case of the local single output approach, the partitioning is more fine-grained as it can be different from one row or column to another. However in this case, each tree gives an interpretable characterization of the nodes which are connected to the node from which the tree was built.

When using ensembles, the global approach provides a global ranking of all features from the most to the less relevant. The local multiple output approach provides two separate rankings, one for the row features and one for the column features and the local single output approach gives a separate ranking for each node. All variants are therefore complementary from an interpretability point of view.

## 3 Experiments

In this section, we carried out a large scale empirical evaluation of the different methods described in Section 2.2 on six real biological networks, three homogeneous graphs and three bipartite graphs. Results on four additional (drug–protein) networks can be found in the ESI.† Our goal with these experiments is to assess the relative performances of the different approaches and to give an idea of the performance one could expect from these methods on biological networks of different nature. Section 3.4 provides a comparison with the existing methods from the literature.

### 3.1 Datasets

The first three networks correspond to homogeneous undirected graphs and the last three to bipartite graphs. The main characteristics of the datasets are summarized in Table 1. The adjacency matrices used in the experiments, the lists of nodes and lists of features can be downloaded at ; http://www.montefiore.ulg.ac.be/schrynemackers/datasets.html.

**Table 1:** Summary of the six datasets used in the experiments

	Network	Network size	Number of edges	Number of features
Homogen. networks	PPI	984 × 984	2438	325
	EMAP	353 × 353	1995	418
	MN	668 × 668	2782	325

Bipartite networks	ERN	154 × 1164	3293	445/445
	SRN	113 × 1821	3663	9884/1685
	DPI	1862 × 1554	4809	660/876

#### 3.1.1 Protein–protein interaction network (PPI)

This network^26^ has been compiled from 2438 high confidence interactions highlighted between 984 *S. cerivisiae* proteins. The input features used for the predictions are a set of expression data, phylogenetic profiles and localization data that totalizes 325 features. This dataset has been used in several studies before.^13,14,27^

#### 3.1.2 Genetic interaction network (EMAP)

This network^28^ contains 353 *S. cerivisiae* genes connected with 1995 negative epistatic interactions. Inputs^29^ consist of measures of growth fitness of yeast cells relative to deletion of each gene separately, and in 418 different environments.

#### 3.1.3 Metabolic network (MN)

This network^30^ is composed of 668 *S. cerivisiae* enzymes connected by 2782 edges. There is an edge between two enzymes when these two enzymes catalyse successive reactions. The input feature vectors are the same as those used in the PPI network.

#### 3.1.4 *E. coli* regulatory network (ERN)

This bipartite network^31^ connects transcription factors (TF) and genes of *E. coli*. It is composed of 1164 genes regulated by 154 TF. There is a total of 3293 interactions. The input features^31^ are 445 expression values.

#### 3.1.5 *S. cerevisiae* regulatory network (SRN)

This network^32^ connects TFs and their target genes from *E. coli*. It is composed of 1855 genes regulated by 113 TFs and totalizing 3737 interactions. The input features are 1685 expression values.^33–36^ For genes, we concatenated motifs features^37^ to the expression values.

#### 3.1.6 Drug–protein interaction network (DPI)

This network^38^ is related to humans and connects a drug with a protein when the drug targets the protein. This network holds 4809 interactions involving 1554 proteins and 1862 drugs. The input features are a binary vectors coding for the presence or absence of 660 chemical substructures for each drug, and the presence or absence of 876 PFAM domains for each protein.^38^

### 3.2 Protocol

In our experiments, LS × LS performances in each network are measured by 10 fold cross-validation (CV) across the pairs of nodes, as illustrated in Fig. 2B. For robustness, results are averaged over 10 runs of 10 fold CV. LS × TS, TS × LS and TS × TS predictions are assessed by performing 10 times 10 fold CV across the nodes, as illustrated in Fig. 2C. The different algorithms return class conditional probability estimates. To assess our models independent of a particular choice of discretization threshold *P*_th_ on these estimates, we vary this threshold and output in each case for the resulting precision–recall curve and the resulting ROC curve. Methods are then compared according to the total area under these curves, denoted AUPR and AUROC respectively (the higher the AUPR and the AUROC, the better), averaged over the 10 folds and the 10 CV runs. For all our experiments, we use ensembles of 100 extremely randomized trees with default parameter setting.^22^

As highlighted by several studies,^39^ in biological networks, nodes of high degrees have a higher chance to be connected to any new node. In our context, this means that we can expect that the degree of a node will be a good predictor to infer new interactions involving this node. We want to assess the importance of this effect and provide a more realistic baseline than the usual random guess performance. To reach this goal, we evaluate the AUROC and AUPR scores when using the sum of the degrees of each node in a pair to rank LS × LS pairs and when using the degree of the nodes belonging to the LS to rank TS × LS or LS × TS pairs. AUROC and AUPR scores will be evaluated using the same protocol as hereabove. As there is no information about the degrees of nodes in TS × TS pairs, we will use random guessing as a baseline for the scores of these predictions (corresponding to an AUROC of 0.5 and an AUPR equal to the proportion of interactions among all nodes pairs).

### 3.3 Results

We discuss successively the results on the three homogeneous networks and then on the three bipartite networks.

#### 3.3.1 Homogeneous graphs

**Fig. 7:**
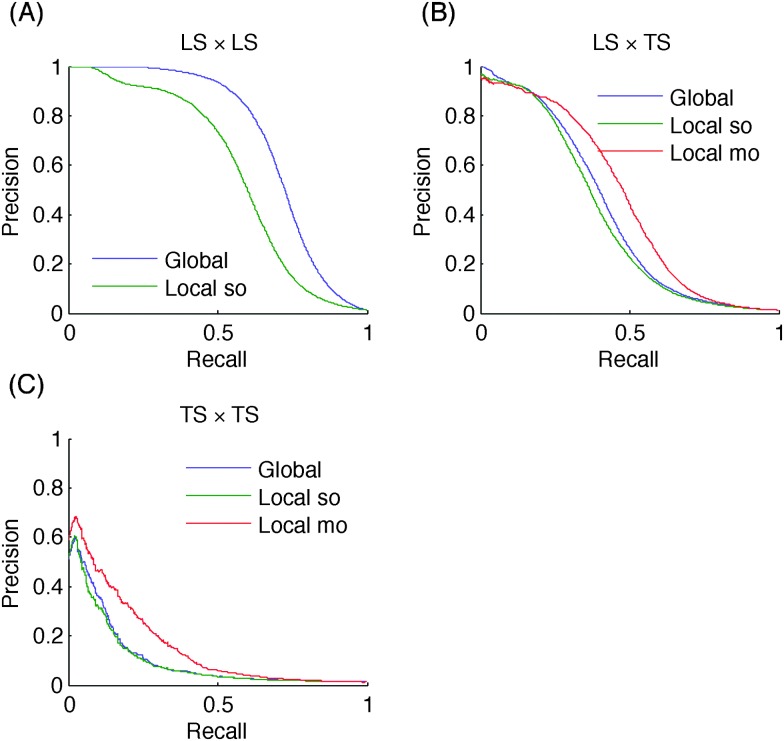
Precision–recall curves for the metabolic network: higher the number of nodes of a pair present in the learning set, better will be the prediction for this pair.

AUPR and AUROC values are summarized in Table 2 for the three methods: global, local single output, and local multiple output. The last row on each dataset is the baseline result obtained as described in Section 3.2. Fig. 7 shows the precision–recall curves obtained by the different approaches on MN, for the three different protocols. Similar curves for the two other networks can be found in the ESI.†

**Table 2:** Areas under curves for homogeneous networks

		Precision–recall (AUPR)			ROC (AUC)		
		LS × LS	LS × TS	TS × TS	LS × LS	LS × TS	TS × TS
PPI	Global	0.41	0.22	0.10	0.88	0.84	0.76
	Local so	0.28	0.21	0.11	0.85	0.82	0.73
	Local mo	—	0.22	0.11	—	0.83	0.72
	Baseline	0.13	0.02	0.00	0.73	0.74	0.50

EMAP	Global	0.49	0.36	0.23	0.90	0.85	0.78
	Local so	0.45	0.35	0.24	0.90	0.84	0.79
	Local mo	—	0.35	0.23	—	0.85	0.80
	Baseline	0.30	0.13	0.03	0.87	0.80	0.50

MN	Global	0.71	0.40	0.09	0.95	0.85	0.69
	Local so	0.57	0.38	0.09	0.92	0.83	0.68
	Local mo	—	0.45	0.14	—	0.85	0.71
	Baseline	0.05	0.04	0.01	0.75	0.70	0.50

In terms of absolute AUPR and AUROC values, LS × LS pairs are clearly the easiest to predict, followed by LS × TS pairs and TS × TS pairs. This ranking was expected from previous discussions. Baseline results in the case of LS × LS and LS × TS predictions confirm that node degrees are very informative: baseline AUROC values are much greater than 0.5 and baseline AUPR values are also significantly higher than the proportion of interactions among all pairs (0.005, 0.03, and 0.01 respectively for PPI, EMAP, and MN), especially in the case of LS × LS predictions. Nevertheless, our methods are better than these baselines in all cases. On the EMAP network, the difference in terms of AUROC is very slight but the difference in terms of AUPR is important. This is typical of highly skewed classification problems, where precision–recall curves are known to give a more informative picture of the performance of an algorithm than ROC curves.^40^

All tree-based approaches are very close on LS × TS and TS × TS pairs but the global approach has an advantage over the local one on LS × LS pairs. The difference is important on the PPI and MN networks. For the local approach, the performance of single and multiple output approaches are indistinguishable, except with the metabolic network where the multiple output approach gives better results. This is in line with the better performance of the global *versus* the local approach on this problem, as indeed both the global and the local multiple output approaches grow a single model that can potentially exploit correlations between the outputs. Notice that the multiple output approach is not feasible when we want to predict LS × LS pairs, as we are not able to deal with missing output values in multiple output decision trees.

#### 3.3.2 Bipartite graphs

**Fig. 8:**
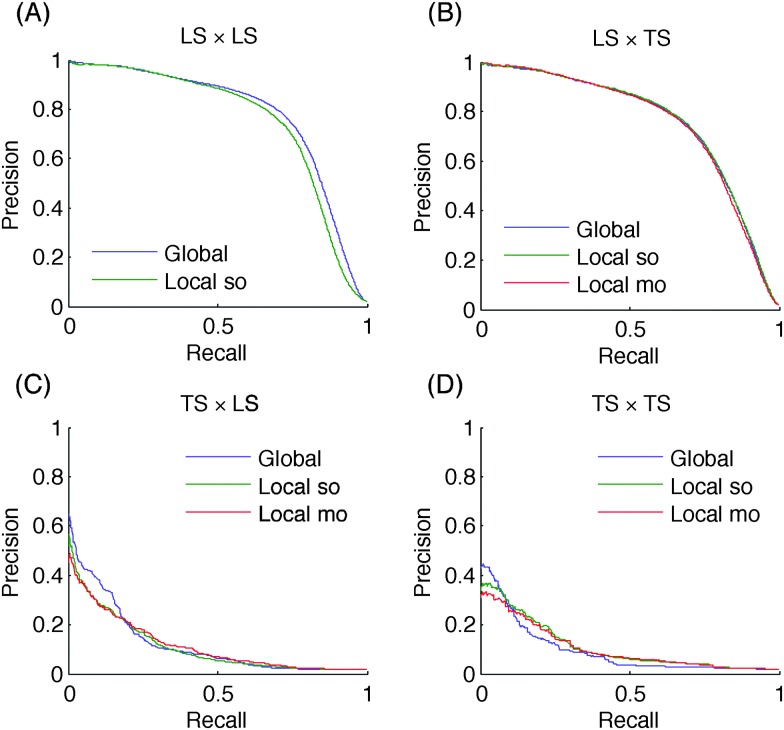
Precision–recall curves for the *E. coli* regulatory network (TF *vs.* genes): a prediction is easier to do if the TF belongs to the learning set than if the gene belongs to.

AUPR and AUROC values are summarized in Table 3 (see the ESI† for additional results on four DPI subnetworks). Fig. 8 shows the precision–recall curves obtained by the different approaches on ERN for the four different protocols. Curves for the 6 other networks can be found in the ESI.† 10 times 10-fold CV was used as explained in Section 3.2. Nevertheless, two difficulties appeared in the experiments performed on the DPI network. First, the dataset is larger than the others, and the 10-fold CV was replaced by 5-fold CV to reduce the computational space and time burden. Second, the feature vectors are binary and the randomization of the threshold (in Extra-tree algorithm) cannot lead to diversity between the different trees of the ensemble. So we used bootstrapping to generate the training set of each tree.

**Table 3:** Areas under curves for bipartite networks

		Precision–recall (AUPR)				ROC (AUC)			
		LS × LS	LS × TS	TS × LS	TS × TS	LS × LS	LS × TS	TS × LS	TS × TS
ERN (TF–gene)	Global	0.78	0.76	0.12	0.08	0.97	0.97	0.61	0.64
	Local so	0.76	0.76	0.11	0.10	0.96	0.97	0.61	0.66
	Local mo	—	0.75	0.09	0.09	—	0.97	0.61	0.65
	Baseline	0.31	0.30	0.02	0.02	0.86	0.87	0.52	0.50

SRN (TF–gene)	Global	0.23	0.27	0.03	0.03	0.84	0.84	0.54	0.57
	Local so	0.20	0.25	0.02	0.03	0.80	0.83	0.53	0.57
	Local mo	—	0.24	0.02	0.03	—	0.83	0.53	0.57
	Baseline	0.06	0.06	0.03	0.02	0.79	0.78	0.51	0.50

DPI (drug–protein)	Global	0.14	0.05	0.11	0.01	0.76	0.71	0.76	0.67
	Local so	0.21	0.11	0.08	0.01	0.85	0.72	0.72	0.57
	Local mo	—	0.10	0.08	0.01	—	0.72	0.71	0.60
	Baseline	0.02	0.01	0.01	0.01	0.82	0.63	0.68	0.50

Like for the homogeneous networks, higher the number of nodes of a pair present in the learning set, better are the predictions, *i.e.*, AUPR and AUROC values are significantly decreasing from LS × LS to TS × TS predictions. On the ERN and SRN networks, performances are very different for the two kinds of LS × TS predictions that can be defined, with much better results when generalizing over genes (*i.e.*, when the TF of the pair is in the learning sample). On the other hand, on the DPI network, both kinds of LS × TS predictions are equally well predicted. These differences are probably due in part to the relative numbers of nodes of both kinds in the learning sample, as there are much more genes than TFs on ERN and SRN and a similar number of drugs and proteins in the DPI network. Differences are however probably also related to the intrinsic difficulty of generalizing over each node family, as on the four additional DPI networks (see the ESI†), generalization over drugs is most of the time better than generalization over proteins, irrespective of the relative numbers of drugs and proteins in the training network. Results are most of the time better than the baselines (based on nodes degrees for LS × LS and LS × TS predictions and on random guessing for TS × TS predictions). The only exceptions are observed when generalizing over TFs on SRN and when predicting TS × TS pairs on SRN and DPI.

The three approaches are very close to each other. Unlike on homogeneous graphs, there is no strong difference between the global and the local approach on LS × LS predictions: it is slightly better in terms of AUPR on ERN and SRN but worse on DPI. The single and multiple output approaches are also very close, both in terms of AUPR and AUROC. Similar results are observed on the four additional DPI networks.

### 3.4 Comparison with related works

In this section, we compare our methods with several other network inference methods from the literature. To ensure a fair comparison and avoid errors related to the reimplementation and tuning of each of these methods, we choose to rerun our algorithms in similar settings as in related papers. All comparison results are summarized in Table 4 and discussed below.

**Table 4:** Comparison with related works on the different networks

Publication	DB	Protocol	Measures	Their results	Our results
Ref. 2	PPI	LS × TS, 5CV	AUPR	0.25	0.21
	MN			0.41	0.43
Ref. 14	PPI	LS × TS, 10CV	AUPR/ROC	0.18/0.91	0.22/0.84
		TS × TS		0.09/0.86	0.10/0.76
	MN	LS × TS		0.18/0.85	0.45/0.85
		TS × TS		0.07/0.72	0.14/0.71
Ref. 3	ERN	LS × TS, 3CV	Recall 60/80	0.44/0.18	0.38/0.15
Ref. 38	DPI	LS × LS, 5CV	AUROC	0.75	0.88
Ref. 41	DPI	LS × LS, 5CV	AUROC	0.87	0.88
		LS × TS & TS × LS		0.74	0.74

#### 3.4.1 Homogeneous graphs

A local approach with support vector machines was developed to predict the PPI and MN networks^2^ and was showed to be superior to several previous works^13,27^ in terms of performance. The authors only consider LS × TS predictions and used 5-fold CV. Although they exploited yeast-two-hybrid data as additional features for the prediction of the PPI network, we obtain very similar performances with the local multiple output approach (see Table 4). Another method^14^ that uses ensembles of output kernel trees also infers the MN and PPI networks with the same input data. With the global approach, we obtain similar or inferior results in terms of AUROC but much better results in terms of AUPR, especially on the MN data.

#### 3.4.2 Bipartite graphs

SVM have been used to predict ERN with the local approach,^3^ focusing on the prediction of interactions between known TFs and new genes (LS × TS). Authors evaluated their performances by the precision at 60% and 80% recall, respectively, estimated by 3-fold CV (ensuring that all genes belonging to the same operon are always in the same fold). Our results with the same protocol (and the local multiple output variant) are very close although slightly less good. The DPI network was predicted using sparse canonical correspondence analyze (SCCA)^38^ and with the global approach and L_1_ regularized linear classifiers^41^ using as input features all possible products of one drug feature and one protein feature. Only LS × LS predictions are considered in the first paper, while the second one differentiates “pair-wise CV” (our LS × LS predictions) and “block-wise CV” (our LS × TS and TS × LS predictions). As shown in Table 4, we obtain better results than SCCA and similar results as in L_1_ SVM. Additional comparisons are presented in the ESI† on the four DPI subnetworks.

Globally, these comparisons show that tree-based methods are competitive on all six networks. Moreover, it has to be noticed that (1) no other method has been tested over all these problems, and (2) we have not tuned any parameters of the Extra-trees method. Better performances could be achieved by changing, for example, the randomization scheme,^7^ the feature selection parameter *K*, or the number of trees.

## 4 Discussion

We explored tree-based ensemble methods for biological network inference, both with the local approach, which trains a separate model for each network node (single output) or each node family (multiple output), and with the global approach, which trains a single model over pairs of nodes. We carried out experiments on ten biological networks and compared our results with those from the literature. These experiments show that the resulting methods are competitive with the state of the art in terms of predictive performance. Other intrinsic advantages of tree-based approaches include their interpretability, through single tree structure and ensemble-derived feature importance scores, as well as their almost parameter free nature and their reasonable computational complexity and storage requirement.

The global and local approaches are close in terms of accuracy, except when we predict LS × LS interactions where the global approach gives almost always better predictions. The local multiple output method has the advantage to provide less complex models and requires less memory and training time. All approaches remain however interesting because of their complementarity in terms of interpretability.

As two side contributions, we extended the local approach for the prediction of edges between two unseen nodes and proposed the use of multiple output models in this context. The two-step procedure used to obtain this kind of predictions provides similar results as the global approach, although it trains the second model on the first model's predictions. It would be interesting to investigate other prediction schemes and evaluate this approach in combination with other supervised learning methods such as SVMs.^20^ The main benefits of using multiple output models is to reduce model sizes and potentially computing times, as well as to reduce variance, and therefore improving accuracy, by exploiting potential correlations between the outputs. It would be interesting to apply other multiple output or multi-label SL methods^42^ within the local approach.

We focused on the evaluation and comparison of our methods on various biological networks. To the best of our knowledge, no other study has considered simultaneously as many of these networks. Our protocol defines an experimental testbed to evaluate new supervised network inference methods. Given our methodological focus, we have not tried to obtain the best possible predictions on each and every one of these networks. Obviously, better performances could be obtained in each case by using up-to-date training networks, by incorporating other feature sets, and by (cautiously) tuning the main parameters of tree-based ensemble methods. Such adaptation and tuning would not change however our main conclusions about relative comparisons between methods.

A limitation of our protocol is that it assumes the presence of known positive and negative interactions. Most often in biological networks, only positive interactions are recorded, while all unlabeled interactions are not necessarily true negatives (a notable exception in our experiments is the EMAP dataset). In this work, we considered that all unlabeled examples are negative examples. It was shown empirically and theoretically that this approach is reasonable.^43^ It would be interesting nevertheless to design tree-based ensemble methods that explicitly takes into account the absence of true negative examples.^44^
