# Supplementary material for: Classifying pairs with trees for supervised biological network inference
Source: Mol Biosyst. 2015 May 26;11(8):2116–25. doi: 10.1039/c5mb00174a (PMC4601289; doi:10.1039/c5mb00174a)
Supplement: Supplementary file 1 [file MB-011-C5MB00174A-s001.pdf]

Electronic Supplementary Material (ESI) for Molecular BioSystems.
This journal is © The Royal Society of Chemistry 2015

Classifying pairs with trees for supervised biological network inference

Supplementary Information

Contents

1 Implementation and computational issues **2**

2 Supplementary performance curves **2**

2.1 Two homogeneous and two bipartite graphs 2

2.2 Four kinds of drug-protein interaction networks 2

3 Illustration of interpretability of trees **7**

3.1 Interpretability of single decision trees 9

3.1.1 Local approach with two multiple-output trees 9

3.1.2 Global approach with one single-output tree 12

3.2 Clustering with ensembles of trees 14

3.2.1 Partitioning the matrix 14

3.2.2 Characterizing the regions 17

3.2.3 Network of features 19

3.3 Pair-based feature ranking 20

1 Implementation and computational issues

In principle, since tree building is a batch algorithm, the global approach requires to generate the full sample of all pairs, which may be very prohibitive for graphs defined on a large number of nodes (e.g., the PPI network used in our experiments contains about 1000 nodes that lead to about 1 millions pairs described by 650 attributes). Fortunately, since the tree building method goes through the input features one by one, one can separately search for the best split on features relative to nodes in U_r and on features relative to nodes in U_c, which does not require to generate explicitly the full data matrix.[1] This is an important advantage with respect to kernel-based methods that typically requires to handle explicitly a $N_rN_c \times N_rN_c$ Gram matrix. Since tree growing is order $O(N\log(N))$ for a training sample of size N, the computational complexity of the whole procedure however remains $O(N_cN_r(\log(N_c) + \log(N_r)))$. The complexity of the trees (measured by the total number of nodes) is at worst $O(N_cN_r)$ (corresponding to a fully developed tree) but in practice it is related to the number of positive interactions in the training sample, which is typically much lower than N_cN_r.

The computational complexity of the local approach is the same as the computational complexity of the global approach, i.e. $O(N_cN_r\log(N_r) + N_rN_c\log(N_c))$. Indeed, in the single output approach, N_c and N_r models need to be constructed respectively from N_r samples and N_c samples each. In the multiple output case, only two models are constructed from N_r and N_c samples respectively, but the multiple output variant needs to go through all outputs at each tree node, which multiplies complexity by respectively N_r and N_c for these two models. However, at worst, the complexity of the model is $O(N_cN_r)$ for the single output approach and $O(N_r + N_c)$ for the multiple output approach, which potentially gives an important advantage along this criterion for the multiple output method.

2 Supplementary performance curves

In this Section, we first present several precision-recall curves, for which the area under the curve are presented in the main paper. Second we present the results obtained on four other drug-protein interaction networks.

2.1 Two homogeneous and two bipartite graphs

Figures S1 and **S2** show the precision-recall curves obtained by the different approaches, respectively on PPI and EMAP (homogeneous graphs), for the three different protocols. **Figures S3** and **S4** show the curves obtained by the approaches, respectively on SRN and DPI (bipartite graphs), for the four different protocols.

2.2 Four kinds of drug-protein interaction networks

[7] proposed four different drug-protein interaction networks in which proteins belong to four pharmaceutically useful classes: enzymes (DPI-E), ion channels (DPI-I), G-protein-coupled receptors (DPI-G) and nuclear receptors (DPI-N). The input features for proteins are similarity with all proteins in terms of sequence and the input features for drugs are similarity with all drugs in terms of chemical structure [7]. The number of drugs in these networks are respectively 445, 210, 223 and 54, the number of proteins are 664, 204, 95 and 26 and the number of interactions are 2926, 1476, 635 and 90 (See **Table S1**).

[1] $U_r = \{n_r^1, \dots, n_r^{N_{U_r}}\}$ and $U_c = \{n_c^1, \dots, n_c^{N_{U_c}}\}$ are the two finite sets of nodes.

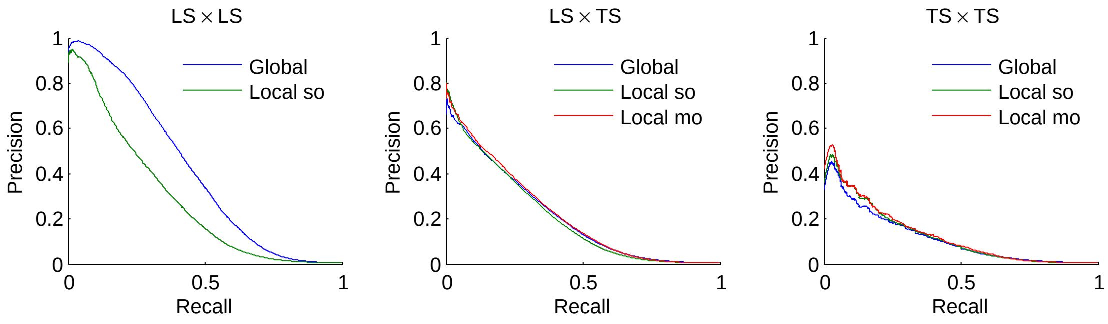

Figure S1: Precision-recall curves for PPI network

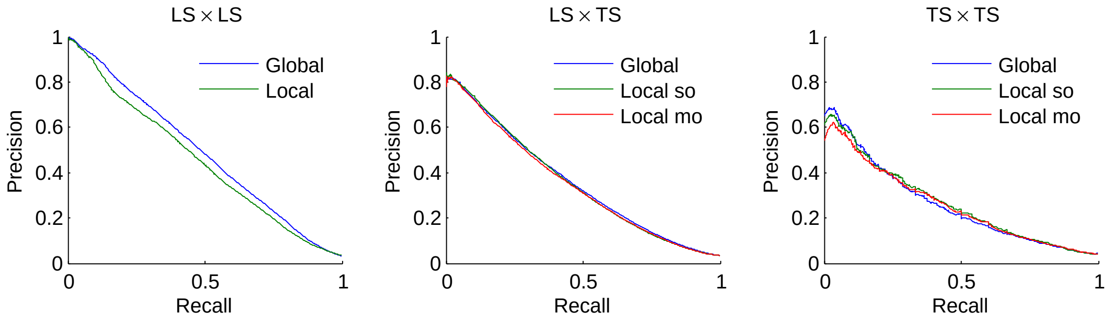

Figure S2: Precision-recall curves for EMAP network

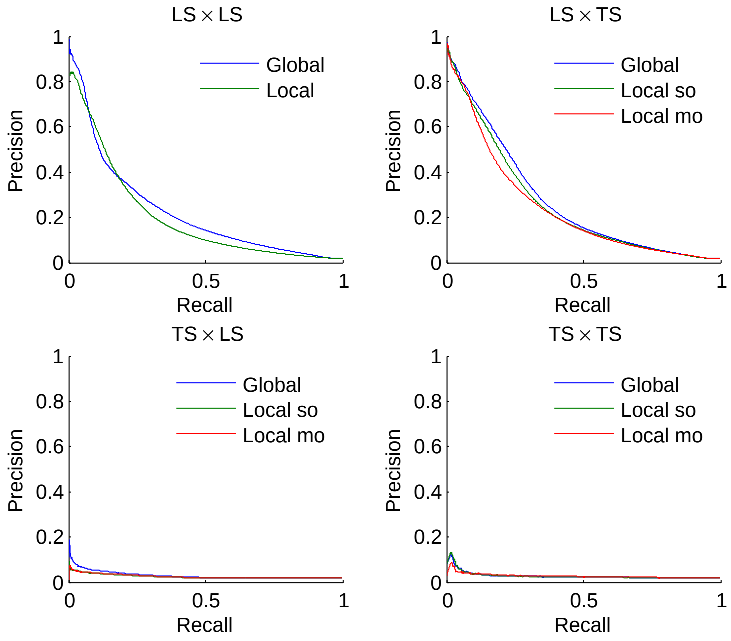

Figure S3: Precision-recall curves for S.cerevisiae regulatory network (TF vs genes)

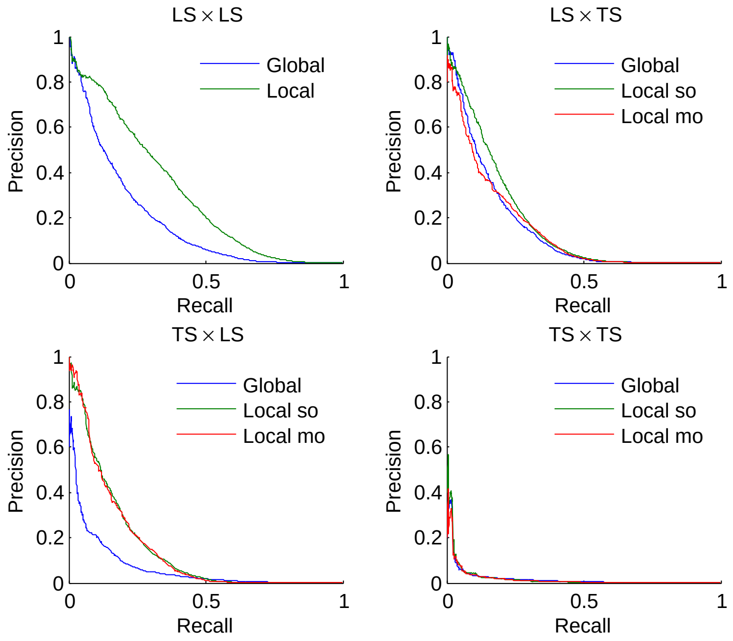

Figure S4: Precision-recall curves for drug-protein interaction network

Areas under precision-recall and ROC curves for the four networks are presented in **Table S2**, while the curves are presented in **Figures S5** to **S8**.

Comparison with literature. [7] and [1] use SVM to predict the four classes of drug-protein interaction network. The first one used a kernel regression-based method (KRM): a global approach in which they integrated the chemical and genomic spaces into a unified space. The second one used bipartite local models (BLM) and then did not predict $TS \times TS$ interactions. We compared the AUPR of these two methods with ours, in a 10 times 10-fold CV, in the following Table. Extra-Trees (E-T) is comparable to the other methods, sometimes giving better results (for DPI-I) and sometimes giving less good results (for DPI-N).

[4] developed three different supervised inference methods, which they tested on the four DPI

Table S1: We carry out experiments on four kinds of drug-protein interaction networks, which are differentiated according to the class the proteins belong. The four classes are: enzymes, ion channels, G-protein-coupled receptors and nuclear receptors.

Network	Network size	# edges	# input features
DPI-E	445×664	2926	445/664
DPI-I	210×204	1476	210/204
DPI-G	223×95	635	223/95
DPI-N	54×26	90	54/26

	Precision-Recall				ROC			
	LS-LS	LS-TS	TS-LS	TS-TS	LS-LS	LS-TS	TS-LS	TS-TS
Drug-protein (enzyme) interaction network								
Global	0.86	0.79	0.32	0.21	0.97	0.93	0.83	0.80
Loc. so	0.82	0.79	0.31	0.20	0.96	0.93	0.82	0.79
Loc. mo	-	0.79	0.32	0.21	-	0.93	0.82	0.78
Drug-protein (ion channels) interaction network								
Global	0.85	0.79	0.31	0.21	0.97	0.93	0.78	0.73
Loc. so	0.81	0.80	0.33	0.23	0.97	0.93	0.78	0.74
Loc. mo	-	0.79	0.33	0.22	-	0.93	0.79	0.74
Drug-protein (GPCR) interaction network								
Global	0.67	0.53	0.32	0.16	0.95	0.85	0.86	0.81
Local so	0.60	0.53	0.33	0.18	0.95	0.84	0.85	0.80
Local mo	-	0.51	0.31	0.16	-	0.84	0.85	0.81
Drug-protein (nuclear receptors) interaction network								
Global	0.45	0.29	0.35	0.13	0.84	0.60	0.79	0.66
Local so	0.43	0.27	0.36	0.12	0.86	0.59	0.80	0.65
Local mo	-	0.27	0.35	0.12	-	0.59	0.80	0.66

Table S2: Areas under curves for the four drug-protein interaction networks.

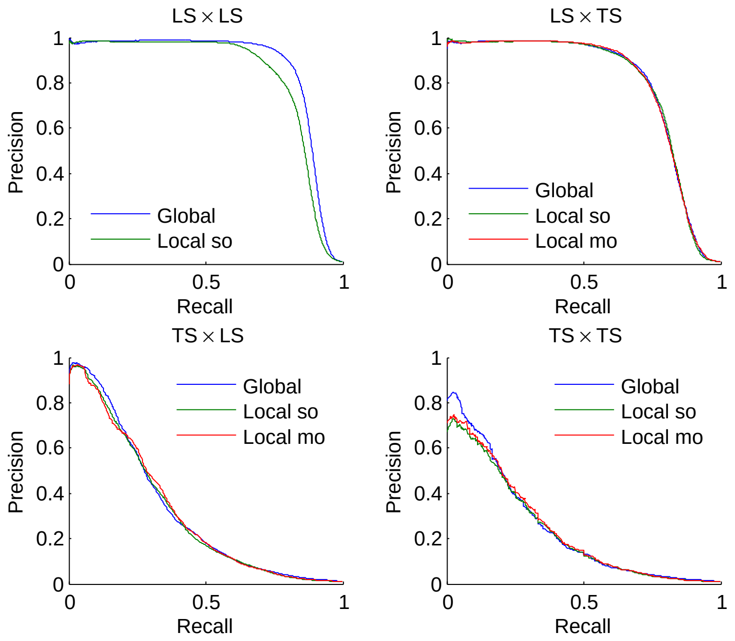

Figure S5: Precision-recall curves for drug-protein (enzymes) interaction network

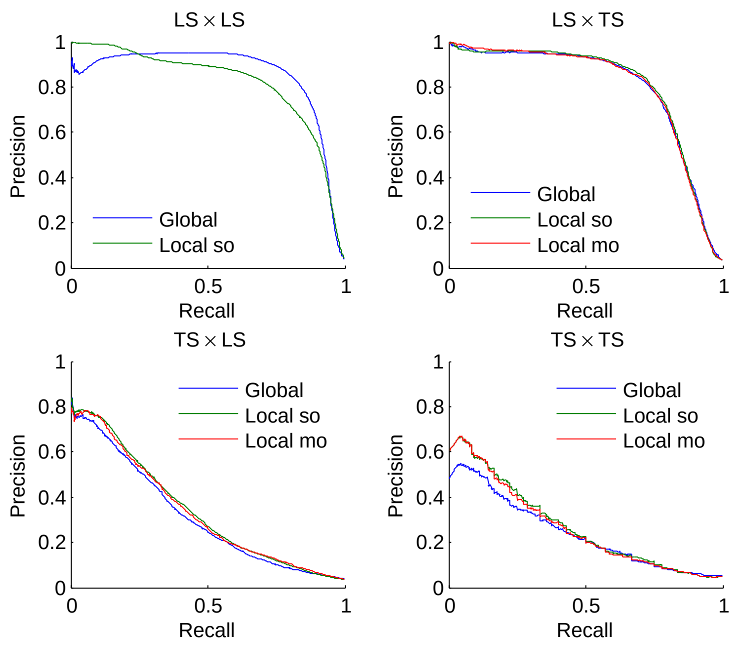

Figure S6: Precision-recall curves for drug-protein (ion channels) interaction network

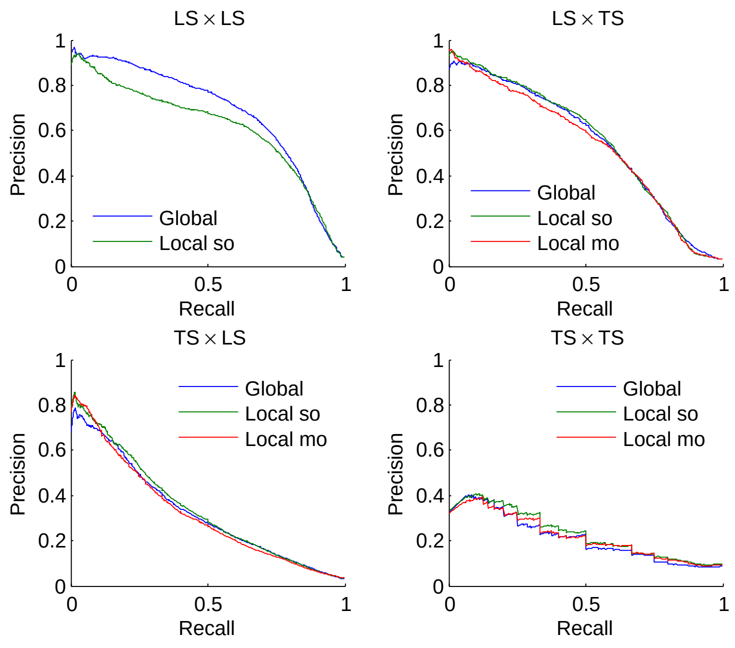

Figure S7: Precision-recall curves for drug-protein (GPCR) interaction network

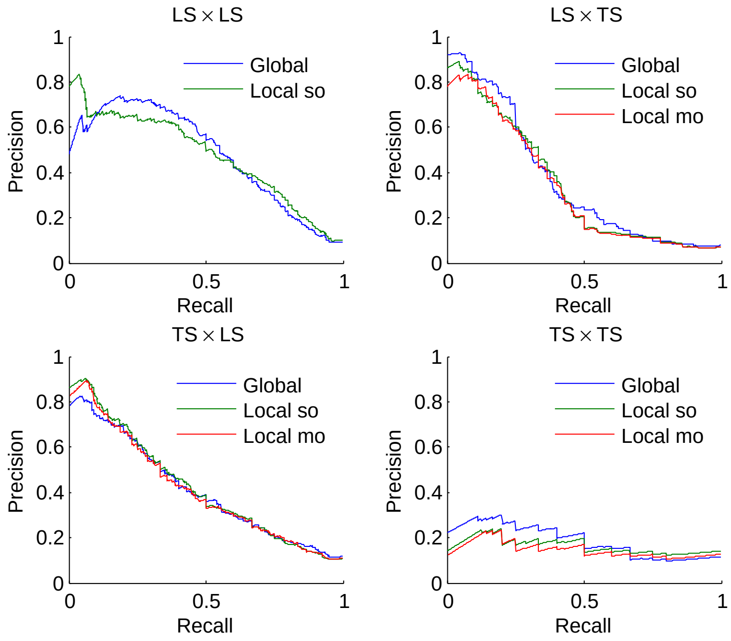

Figure S8: Precision-recall curves for drug-protein (nuclear receptors) interaction network

datasets. The methods are drug-based similarity inference (DBSI), target-based similarity inference (TBSI) and network-based inference (NBI). The last one only use network topology similarity to to infer new targets for known drugs. NBI gives the best performance of the three but has the disadvantage to only be able to predict $LS \times LS$ pairs. Extra-Trees give better or equal results than these three methods, when doing 10 times 10-fold CV. Results are presented in **Table S3**.

3 Illustration of interpretability of trees

One advantage of tree-based methods is their interpretability. In this section we will illustrate this interpretability by carrying out some experiments on a drug-protein interaction network. Note that our goal with these experiments is merely to propose and illustrate several semi-automatic procedures to extract interpretable information from tree-based models. We will not try to assess the biological relevance of our findings.

The dataset we will use for the illustration contains 4809 interactions involving 1862 drugs and 1554 proteins, which gives a proportion of interactions equal to 0.17%. Common chemical substructures are often shared by drugs that bind a given protein, and common function sites (e.g., PFAM domains) are often shared by proteins that are bound by a given drug [6]. Input data used to perform predictions is therefore composed of:

- a binary vector coding for the presence or the absence of 660 chemical substructures for each drug,

- a binary vector coding for the presence or the absence of 876 PFAM domains for each protein.

Table S3: We compared the results obtained with our method (E-T) with several results from the literature.

	Method	Precision-Recall			Method	ROC
		LS-LS	LS-TS	TS-LS		LS-LS
DPI-E	KRM[7]	0.83	0.81	0.38	DBSI[4]	0.78
	BLM[1]	0.83	0.81	0.39	TBSI[4]	0.90
					NBI[4]	0.97
	E-T	0.87	0.79	0.32		0.97
DPI-I	KRM	0.76	0.81	0.31	DBSI	0.71
	BLM	0.77	0.80	0.32	TBSI	0.90
					NBI	0.98
	E-T	0.85	0.80	0.34		0.97
DPI-G	KRM	0.67	0.62	0.41	DBSI	0.76
	BLM	0.65	0.55	0.38	TBSI	0.75
					NBI	0.94
	E-T	0.68	0.55	0.34		0.95
DPI-N	KRM	0.74	0.61	0.51	DBSI	0.79
	BLM	0.58	0.35	0.40	TBSI	0.53
					NBI	0.84
	E-T	0.48	0.36	0.42		0.86

We first discuss interpretability in the case of single decision trees built using the local or the global approach (Section 3.1). We then show how to extract information from ensembles, first in the form of biclusters (Section 3.2) and second in the form of pair-based feature rankings (Section 3.3).

3.1 Interpretability of single decision trees

As discussed in Section 3.3.4 of the paper, the local approach with multi-output trees yields a checkerboard structured biclustering of the adjacency matrix, while the global approach yields an unconstrained biclustering of the same matrix. In both cases, each bicluster is defined by a subset of row nodes (drugs) and a subset of column nodes (proteins) and by construction is such that pairs defined by these two subsets are either highly connected or highly disconnected. We illustrate below both methods on the DPI network.

3.1.1 Local approach with two multiple-output trees

With the local approach and multi-output trees, two single trees are built: the first one partitions the row nodes (the drugs) and the other partitions the column nodes (the proteins). The drugs that end in a same leaf form a cluster of drugs, and the proteins that end in a same leaf form a cluster of proteins. If we look at the network through its adjacency matrix, each pair of drug and protein clusters define a rectangular subregion of the adjacency matrix and the rectangular regions corresponding to all pairs can be arranged as a checkerboard (see **Figure 4B**). Each of these rectangular regions, or biclusters, corresponds to a bipartite subnetwork of the global network. Because of the way the two trees are built, we expect that the resulting subnetworks have either significantly many or significantly few drug-protein interactions. Indeed, drugs (resp. proteins) that fall in the same leaf are assumed to have very similar connectivity profiles with all proteins (resp. drugs), i.e., the corresponding rows (resp. columns) in the adjacency matrix are expected to be similar. Putting together these two constraints (similar rows and similar columns), the rectangular subregion of the adjacency matrix defined by a cluster of drugs and a cluster of proteins should thus either contain many 1s or many 0s. Each cluster of drugs/proteins furthermore corresponds to a path in one of the tree, i.e. a conjunction of tests based on the input features. Given the nature of the features for this particular network, clusters of drugs are thus characterized by the absence or presence of some chemical substructures (those that are tested along the tree path), while similarly clusters of proteins are characterized by the absence or presence of some PFAM domains. To understand the model, one should thus look at the subnetworks defined by the two trees that contain a significantly high number of interactions and extract from the tree the chemical substructures and PFAM domains that characterize these subnetworks.

Figure S9 shows the checkerboard partition of the adjacency matrix obtained on the drug-protein interaction network. The grey level of each submatrix represents the ratio of interactions among all pairs in the submatrix. To obtain this partition, we arbitrarily stop splitting a node in each tree if the number of drugs or proteins it contains goes below 10% of the total number of drugs or proteins. This corresponds to using a value of $n_{\mathrm{min}} = 186$ for the drugs and $n_{\mathrm{min}} = 155$ for the proteins. Applying some pruning is necessary to get meaningful clusters. Indeed, if one lets the algorithm grow fully developed trees, most tree leaves, and therefore clusters, will typically contain only one or very few nodes (since most drugs and proteins have different connectivity profiles).

The partition of **Figure S9** highlights the very unbalanced nature of the grown trees. If the trees were balanced, given our choice of n_{min} we would have obtained about 10 clusters/leafs per dimension, while we have obtained much more leaves, in particular in the tree grown for the proteins (52 for the

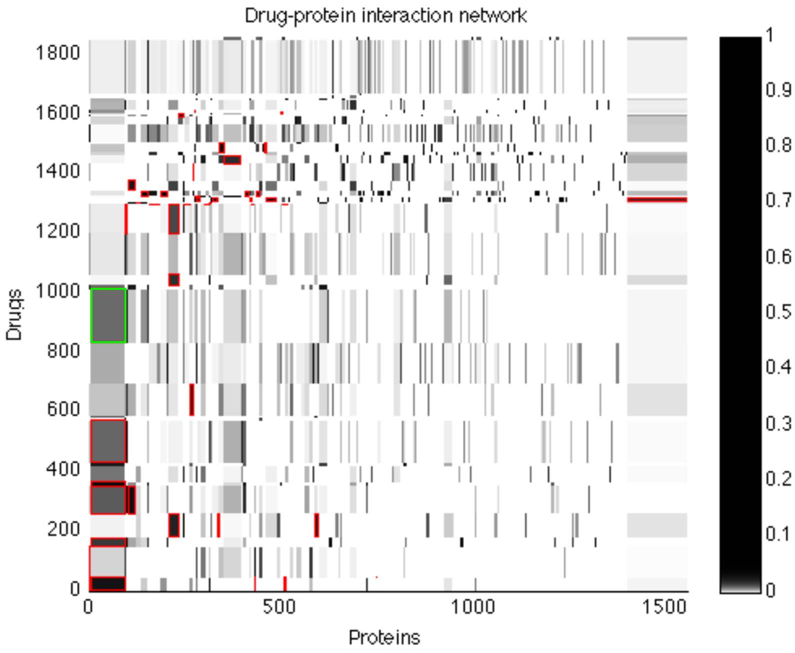

Figure S9: The adjacency matrix of the drug-protein interaction network is partitioned into clusters by the two single multi-output trees. Rows represent drugs, while columns represent proteins. Each pair of clusters is expected to be either very connected, or not. The 47 submatrices with p-values lower than 10^{-7} are highlighted in red. In total, they contain a proportion of interactions equal to 2%.

drugs and 475 for the proteins). This is a consequence of the difficulty of the task: several tree splits only separate a few drugs/proteins from the rest of the drugs/proteins in the corresponding tree node and many splits are thus required to reach the 10% size limit.

We observe also that most subregions contain a large majority of 0s. This is not surprising. Indeed, given the very sparse nature of the network, we did not expect to find subnetworks where all drugs are interacting with all proteins. We nevertheless expect that some of them will contain proportionally more interactions than the global network and some of them proportionally less interactions than the global network. As the goal is mainly to understand what defines the interactions (not the non-interactions), the most interesting subnetworks are those which are proportionally more connected than the global network. To identify them in our illustrative problem, we computed a *p*-value for each subnetwork that measures whether it is significantly connected or not with respect to a randomly selected subnetwork of the same size. This *p*-value can be estimated by random permutations: we randomly draw 10^7 windows of the same size (i.e., with the same numbers of drugs and proteins) in the adjacency matrix, and count how many of these windows contain at least the same number of connections as the target window. By dividing the resulting number by 10^7, we obtain the probability to have a more connected window by chance from the global network. We computed the *p*-values relative to all submatrices on the drug-protein network. The 47 regions that obtained an estimated *p*-value lower than 10^{-7} are highlighted in red in **Figure S9**. These are thus the subregions for which we did not find any random subregions of the same size with an equal number or more connections. In total, these 47 regions contain a proportion of interactions equal to 2%, which is more than 12 times higher than the proportion of interactions in the global network (i.e., 0.17%).

To illustrate the feature-based characterization of the submatrices, let us analyze the significant submatrix with the highest number of interactions. This submatrix is located in the green rectangle in **Figure S9** between the drugs numbered 835 and 1014 and the proteins numbered 6 and 94. **Figure S10** shows a zoom of this submatrix, where interactions are represented by black dots. One counts in this submatrix 152 interactions between 180 drugs and 89 proteins, which gives a proportion of interactions almost 6 times greater than in the global network.

The path in the tree relative to drugs that leads to this submatrix is composed of a succession of tests based on the presence or absence of the following drug substructures (in their order of appearance in the path):

1. SC1C(N)CCCC1
2. $>=$ 1 Co
3. N-S-C:C
4. $>=$ 1 saturated or aromatic nitrogen-containing ring size 8'
5. $>=$ 1 unsaturated non-aromatic carbon-only ring size 10' 'C($\sim$Br)($\sim$H)
6. $>=$ 1 any ring size 7
7. **$>=$ 1 saturated or aromatic carbon-only ring size 6**
8. **C($\sim$C)($\sim$H)($\sim$N)**
9. O-C:C-O-[♯1]
10. N-H
11. Nc1cc(Cl)ccc1
12. O-C:C-O

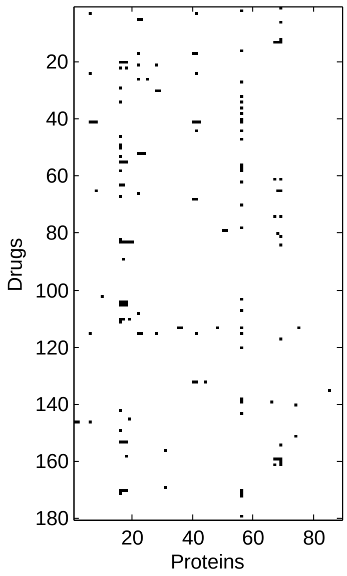

Figure S10: The submatrix having the greatest number of interaction, among those with a p-value smaller than 10^{-7}, is characterized in the text.

In this list, the two substructures in bold are the substructures present in the 180 drugs of the subnetwork, while the others are absent in all of them. Similarly, the path in the tree relative to the proteins is composed of a succession of tests based on the presence or absence of the following three PFAM domains:

1. Eukaryotic-type carbonic anhydrase
2. Oestrogen receptor
3. **7 transmembrane receptor (rhodopsin family)**

The bold PFAM domain is the domain present in the 89 proteins of the subnetwork, while the two others are absent in all of them. The features in these two lists, particularly the bold ones, are expected to explain the significantly high proportion of drug-protein interactions within the cluster: a drug with one or more saturated or aromatic carbon-only ring of size 6 and which contains C(∼C)(∼H)(∼N) as a substructure is more likely to interact with a protein from the rhodopsin family than any random protein.

3.1.2 Global approach with one single-output tree

The global approach with single tree can also be interpreted as carrying out a biclustering of the adjacency matrix. Only one tree is built in this case, which classifies drug-protein pairs. Each leaf of this tree corresponds to a subset of pairs from the learning sample. As features are defined either on drugs or on proteins (not on pairs), each leaf also corresponds to a cluster of drugs and a cluster of proteins and all pairs that involve drugs and proteins from these clusters fall into that leaf. Like for the local approach, each leaf thus also delimits a rectangular subregion of the adjacency matrix and this region is therefore characterized by a path in the tree, and consequently by a conjunction of tests based

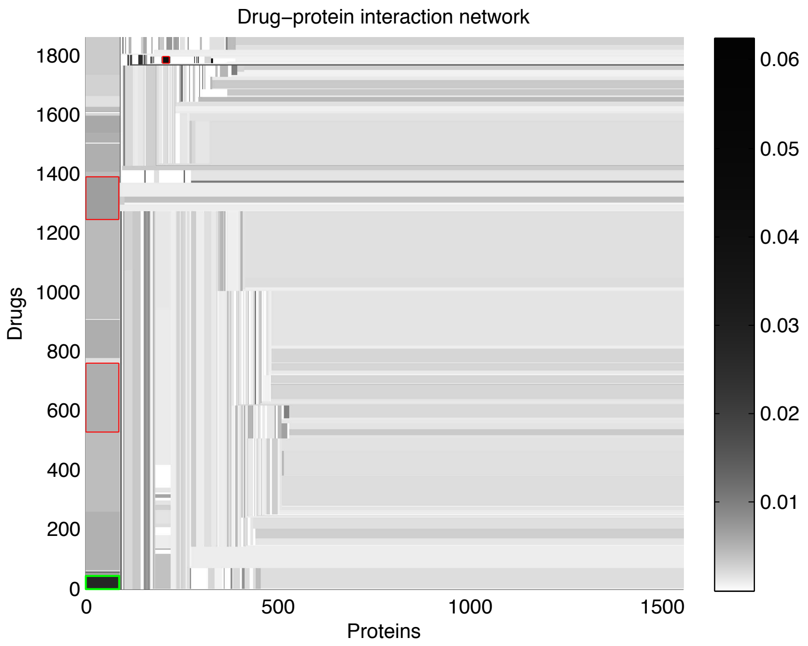

Figure S11: The adjacency matrix of the drug-protein interaction network is partitioned into clusters, due to one global single tree. Rows represent drugs, while columns represent proteins. The pairs of each clusters are expected to be either very connected, or not. The four submatrices with p-values lower than 10^{-7} are red surrounded, and have a proportion of interaction equal to 0.9%.

on drug or protein features (see **Figure 5A**). Given the way the tree is built, each leaf subregion is also expected to contain either significantly many or significantly few drug-protein interactions.

Figure S11 shows the resulting partitioned adjacency matrix, where again the grey level of each submatrix represents the percentage of interactions it contains. To obtain this partition, we arbitrarily stopped splitting a node of the tree if the number of pairs within it went below 1% of the total number of pairs (i.e., $n_{min} = 28935$). As a result, the matrix is divided into 691 rectangular subregions. Note that in the case of the global approach, it is not possible anymore to represent the resulting partitioning of the adjacency matrix as a checkerboard, where each row corresponds to a unique proteins and each column to a unique drug. Indeed, each new split cuts the corresponding subregion of the adjacency matrix either horizontally (when it is based on a drug feature) or vertically (when it is based on a protein feature) but there is no guarantee that the left and right successors of a split will be cut in the same way. However, given the hierarchical nature of the partitioning, the rectangular subregions defined by all tree leaves can be arranged so that they exactly cover the adjacency matrix. This is the representation used in **Figure S11**, which is obtained by recursively reordering the rows and columns of the subregions according to the tree splits.

As in the case of the local approach, we are mainly interested in the leaves corresponding to drugs and proteins that are significantly highly connected. We therefore again computed for each submatrix an interaction p-value using the same permutation scheme as for the local approach. Subregions with a p-value smaller than 10^{-7} are highlighted in green in **Figure S11**. We only found four regions that meet

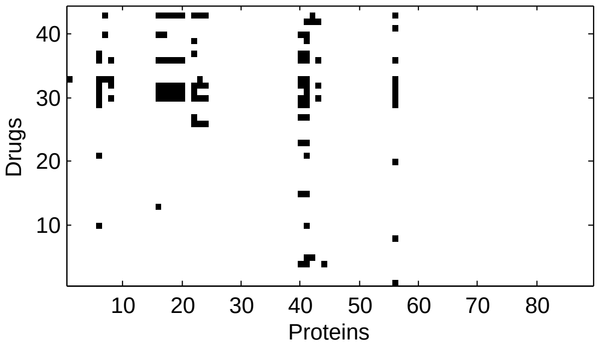

Figure S12: One of the four submatrices that got a p-value lower than 10^{-7}, and which is characterized in the text.

this criterion, which is much fewer than the 47 regions found with the local approach. We also observed a very important difference in terms of the number of subregions defined by both methods, despite that fact that their n_{min} values were matched: 691 subregions are found with the global approach and 24700 subregions with the local one. These differences can be explained by the more flexible nature of the global approach that can define unconstrained partitions, while the local approach can only define checkerboard-structured partitions.

These four regions of small p-value contain in total 0.9% of all interactions. One of these regions appears to be found also in the clustering obtained with the local approach, and is characterized here below. It is located in bottom left corner of **Figure S11**. A detailed view of this submatrix can be found in **Figure S12**. It counts 109 interactions between 44 drugs and 89 proteins, which gives a proportion of interactions 5 times greater than the proportion in the global network. Analyzing the tree path that leads to the corresponding leaf, this subregion is defined by all drugs that contain SC1C(N)CCCC1 as a chemical substructure and all proteins, from the rhodopsin family, that has the 7 transmembrane receptor PFAM domain.

3.2 Clustering with ensembles of trees

In Section 3.1, we used single trees to partition the adjacency matrix. Yet we know that single trees suffer from high variance and may not be the ideal method to obtain interpretable results. In this section, we propose a way to obtain from an ensemble of trees a clustering of the drugs and proteins as well as a characterization of the corresponding subnetworks in terms of the input features. The procedure is explained and illustrated below using the drug-protein interaction network.

3.2.1 Partitioning the matrix

To obtain a checkerboard partitioning of the adjacency matrix from an ensemble of trees, we propose to proceed as follows. Two ensembles of (100) multi-output trees are grown respectively for the drugs and the proteins using the local approach. From the ensemble relative to the drugs (resp. proteins), one can derive a proximity measure between two drugs (resp. proteins) by counting the number of times the two drugs (resp. proteins) fall in the same leaf over the 100 trees in the ensemble and by normalizing this count by the total number of trees in the ensemble [3]. From this proximity, a distance

between drugs/proteins can be computed as one minus the proximity. Using this distance measure, one can build two distance matrices, one 1862 $\times$ 1862 matrix for the drugs and one 1554 $\times$ 1554 matrix for the proteins and these matrices can be used as inputs of the k-medoids algorithm to obtain a clustering respectively of drugs and proteins. Just as in the local approach with single multi-output trees, these two clusterings then define a checkerboard partitioning of the adjacency matrix. Because the ensemble proximity is such that two drugs (resp. proteins) are close if they have similar connectivity patterns with all proteins (resp. drugs), each subregion of this partitioning should contain either strongly or weakly connected pairs

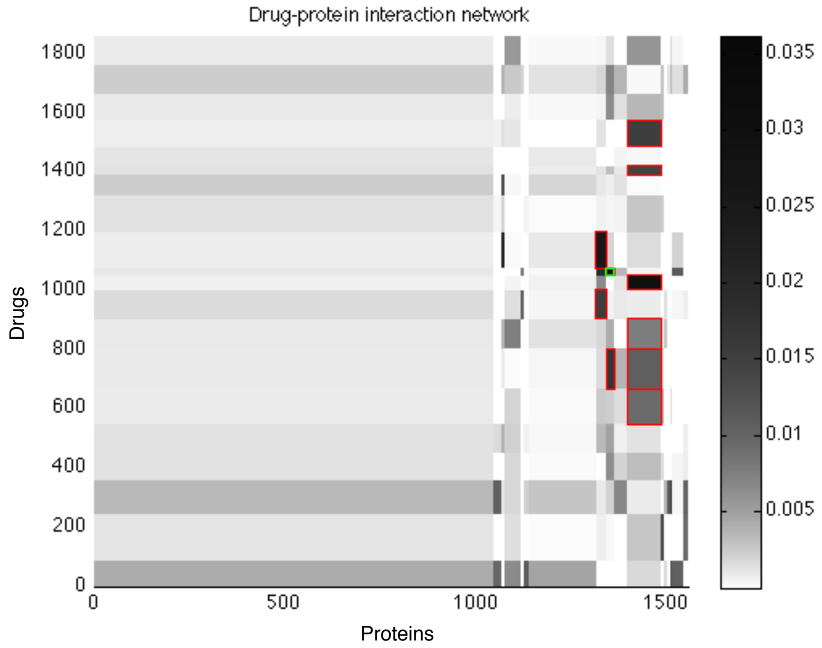

Figure S13: The adjacency matrix of the drug-protein interaction network is partitioned into clusters, using a proximity measure derived from ensembles of multi-output trees. The pairs in each clusters are expected to be either very connected or not. The ten regions highlighted in red are the ones with the smallest interaction p-values estimated by random permutations. Together, they contain a proportion of interactions equal to 1.4%

Figure S13 shows the checkerboard structure obtained with this procedure using the k-medoids algorithm to find 20 clusters for the drugs and 20 clusters for the proteins. Trees were grown using the same n_{min} thresholds as in the experiment of **Figure S9**. The clusters of drugs are almost all of the same size, while clusters of proteins are much more unbalanced in sizes, with some very small (as observed in **Figure S9** with single trees) and some very large clusters.

The grey level of each submatrix represents the ratio of interactions within it. To identify significantly connected submatrices, we computed an interaction p-value for each of them as we did in Section 3.1. The 10 regions corresponding to p-values smaller than 10^{-7} are highlighted in red in **Figure S13**. In total, they contain a proportion of 1.4% interacting pairs, which is more than 8 times higher than the proportion in the global network.

Figure S14 shows a graphical representation of the whole network. The drugs correspond to the

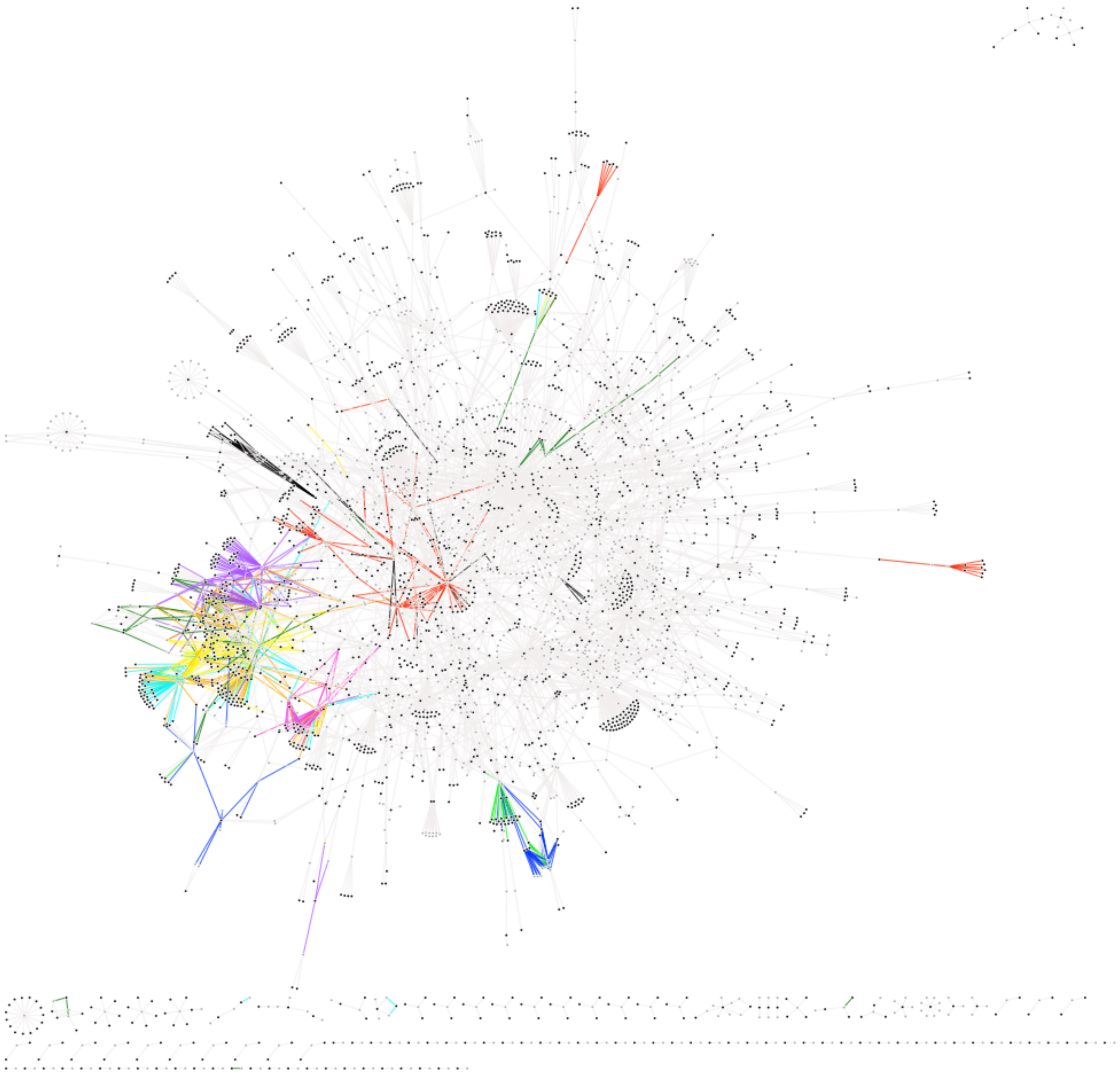

Figure S14: A graphical representation of the drug-protein interaction network. The ten subnetworks with the lowest p-values are highlighted by colored edges.

black nodes and the proteins to the grey nodes. Interactions that are not included in one of the ten submatrices with lower p-values, are represented by light grey edges. The interactions from the ten submatrices are highlighted with different colors. We can see that, mostly, the edges from a same cluster of interactions are located in a same region of the graph, showing that the drugs and the proteins that make it up are indeed highly connected.

Note that the proximity measure proposed above can be shown to be a dot-product into some euclidean space [3]. Indeed, let us encode the ith drug/protein with a vector of the following form:

$$\mathbf{v}_i = [[l^i_{1,1}, l^i_{1,2}, \dots, l^i_{1,n_1}], [l^i_{2,1}, l^i_{2,2}, \dots, l^i_{2,n_2}], \dots, [l^i_{T,1}, l^i_{T,2}, \dots, l^i_{T,n_T}]]^\top \quad (1)$$

where T is the number of trees in the ensemble, n_k is the number of leaves in the kth trees, and $l^i_{k,l}$ is equal to $1/\sqrt{T}$ if drug/protein i falls into leaf k of the lth tree, 0 otherwise. Then, the proximity between two drugs/proteins is equal to the dot-product $\mathbf{v}_i^\top \mathbf{v}_j$ between their corresponding vectors $\mathbf{v}_i$ and $\mathbf{v}_j$ and their distance is the euclidean squared distance between these vectors $||\mathbf{v}_i - \mathbf{v}_j||^2$. An alternative to the application of the k-medoids algorithm used above is thus to apply k-means on this vectorial representation. Finally, note that [5] have proposed another proximity measure that uses the same encoding as in (1) but with $l^i_{k,l}$ taken as $1/\sqrt{TN_{k,l}}$ when drug/protein i falls into the kth leaf of the lth tree, where $N_{k,l}$ is the total number of learning sample examples falling into the same leaf. This proximity measure gives more weights to leaves that contain fewer examples. Both measures coincide in the case of fully grown trees with only one example per leaf.

3.2.2 Characterizing the regions

The clustering proposed above is based on the ensemble of trees and thus the fact that a drug/protein belongs to a specific cluster is a direct consequence of its input feature values. For interpretability reason, it is thus interesting to try to characterize each cluster from the point of the view of the input features. Unlike with single trees (see Section 3.1), this is not as straightforward as looking at the features that are used along some tree path, because all drugs or proteins in a given cluster do not end automatically in the same leaf in a given tree and also because we now have an ensemble of different trees and not a single tree. Feature importance scores as proposed in [2] only give a global measure of the importance of a feature, while we are looking here for a more local cluster-specific characterization.

To answer this question, we propose in this section a way to derive local feature importance scores relative to a subset of objects (drugs or proteins). Given an ensemble of trees and a subset of objects S, local feature importance scores are computed as follows:

- For a given object o in S and a tree $\mathcal{T}$, we compute the importance of a feature X_i, denoted $I(X_i, o, \mathcal{T})$, as the sum of weighted impurity reductions over all nodes in the path traversed by o in $\mathcal{T}$ where X_i is used as the splitting feature. Impurity reductions are computed on the basis of the training sample from which the ensemble was grown.

- These importances are then averaged over all objects in S and all trees in the ensemble to obtain the importance of feature X_i relative to the subset S:

$$I(X_i, S) = \frac{1}{T} \sum_{t=1}^{T} \frac{1}{|S|} \sum_{o \in S} I(X_i, o, \mathcal{T}_t) \quad (2)$$

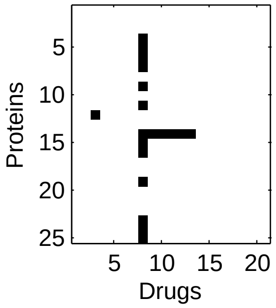

Figure S15: The submatrix having the highest proportion of interactions, among those with a *p*-value lower than 10^{-7} in **Figure S13**. This submatrix is characterized in the text.

Intuitively, a feature will be important according to this modified local measure if it is used in a lot of paths traversed by objects from the subset S and if, in these paths, it leads to important weighted impurity reductions. Note that when S is taken as the whole learning sample, this measure does not coincide with the global feature importance score defined in [2]. This property could have been obtained by summing unweighted instead of weighted impurity reductions in the first step above but we believe that this would have given too much importance to splits at deep nodes in the tree in the case of small subsets of objects S.

Let us illustrate this cluster-specific feature importance measure on the drug-protein interaction network. We focus on the submatrix with the higher proportion of interactions, among the ten submatrices with a *p*-value lower than 10^{-7}. This submatrix is highlighted in green in **Figure S13** and its edges are in light green in **Figure S14**. A detailed view of this submatrix can be found in **Figure S15**. It contains 19 interactions between 25 drugs and 21 proteins, corresponding to 3.6% of interactions. Feature importance scores for the drug and protein features were derived respectively for the subsets of 25 drugs and 21 proteins using the procedure above. Given the multi-output setting, we use the average variance over all outputs as our impurity measure.

392 chemical substructures (drug features) obtained an importance higher than zero for the cluster of 25 drugs. We list here only the ten most important ones (in decreasing order of importance):

1. SC1C(N)CCCC1
2. >= 1 saturated or aromatic nitrogen-containing ring size 8
3. C(∼F)(∼F)
4. N-S
5. S(=O)(=O)
6. O=C-C-C-C-C(=O)-C
7. **C(∼C)(∼C)(∼C)(∼C)**
8. Sc1c(N)cccc1
9. C(∼Br)(∼H)
10. CC1CC(O)CC1

For each substructure in this list, we computed its occurrence frequency in the cluster and in the whole set of drugs. Only one substructure (C(∼C)(∼C)(∼C)(∼C), in bold in the list) appears more frequently in the 25 drugs than in the whole set of drugs.

Concerning protein features, 158 PFAM domains have an importance higher than zero for the cluster of 21 proteins. We list here only the ten most important ones (in decreasing order of importance):

1. Eukaryotic-type carbonic anhydrase
2. **Neurotransmitter-gated ion-channel transmembrane region**
3. 7 transmembrane receptor (rhodopsin family)
4. Animal haem peroxidase
5. **Neurotransmitter-gated ion-channel ligand binding domain**
6. Oestrogen receptor
7. Serum albumin family
8. Serotonin (5-HT) neurotransmitter transporter, N-terminus
9. TspO/MBR family
10. Aminotransferase class I and II

The two bold PFAM domains are present in each of the 21 proteins of the cluster, while the 7 other domains are absent in all of them. These two lists of features, and in particular the ones in bold, are those that characterize the most this highly connected region.

3.2.3 Network of features

In [6], the authors exploited canonical correlation analysis to find associations between protein domains and chemical substructures that are predictive of drug-protein interactions. Each canonical component highlights several such associations and associations corresponding to all canonical components are compiled into a global (bipartite) network. This network contains 30,668 links between chemical substructures and protein domains that represent 5,3% of all possible links that can exist between the 660 chemical substructures and the 876 protein domains. All these links are expected to govern drug-protein interactions.

Similarly, each submatrix found by our method also associates chemical substructures and protein domains, those that appear in the feature importance ranking obtained for both drug and protein clusters. A network similar to [6]'s network can thus be constructed from tree ensembles by linking chemical substructures and protein domains found in the feature importance lists associated to all submatrices containing a significantly high number of interactions. As noted previously, the chemical structures or protein domains with non zero importance for a given cluster are not always present in the drugs and proteins within the cluster. We thus filter the links so as to only keep the ones that involve features that are more present in the drug or proteins of the cluster than in the whole set of drugs or proteins.

We applied this idea from the partitioning found in **Figure S13**. The network built from the ten clusters with the smallest p-values ($< 10^{-7}$) contains 3086 interactions. It corresponds to 0.5% of all possible links between the 660 chemical substructures and the 876 protein domains. From these 3086 interactions, 992 (32%) can also be found in [6]'s network (see **Figure S16**), while 2094 are new. The intersection with [6]'s network is very significant, as assessed with a hypergeometric test (p-value$< 10^{-100}$). The network can obviously be enlarged by considering clusters with p-values higher than 10^{-7}.

As in [6], a weight can be associated to an edge in this network by computing the product of the importance value associated to the chemical substructure and the protein domain that it connects. In

Figure S16, we present the network obtained when keeping only the 17 edges with a weight higher than the arbitrary chosen threshold $6 \cdot 10^{-6}$. From these 17 interactions, 6 can also be found in [6]'s network (intersection p-value$= 1.43 \cdot 10^{-5}$).

3.3 Pair-based feature ranking

A main goal of network inference methods is to rank new pairs from the most likely to the least likely to interact. When using single trees, one gets directly an explanation of the predictions in terms of the features that are tested along the path followed in the tree, either by the pair (in the case of the global approach) or by each of its nodes (in the case of the local approach). With an ensemble of trees, this feature is lost but it is nevertheless possible to obtain a ranking of the features according to their importance for making a specific pair prediction. To obtain such ranking, one can indeed use the local importance measure defined in Equation 2 using as the subset S a singleton containing one node from the pair (in the case of the local approach) or one pair (in the case of the global approach).

Let us illustrate this possibility on the drug-protein interaction network. We ranked the drug-protein pairs with a local approach using single-output tree ensembles. To get a prediction for all pairs of the network, we performed a 5-fold cross-validation on pairs. 8 pairs were predicted with the highest probability to interact (i.e. 1), and they are listed here above:

Drug	Protein	Interaction
5,6,7,8-Tetrahydrobiopterin	NOS3	1
6-Mercaptopurine	IMDH1	1
6-Mercaptopurine	PUR1	1
Epothilone B	TBA2	1
Epothilone B	TBA6	1
Hydrochlorothiazide	CAH4	1
Thioguanine	IMDH2	0
Tretinoin	RXRG	1

Only one pair is actually not interacting, or has not been discovered as interacting yet.

We focus on the interaction between 5,6,7,8-Tetrahydrobiopterin and NOS3 (**Figure S17**). This interaction allows the production of nitric oxide (NO), which is important in regulation of blood pressure and blood flow. For this particular pair, we ranked both chemical substructures and protein domains according to their local importances for predicting this particular pair. To compute these importance scores, we used the two ensembles (one for 5,6,7,8-Tetrahydrobiopterin and one for NOS3) grown from the learning fold (among the five) that did not contain this particular pair.

The 10 more important chemical substructures are the following:

1. **C(~C)(~C)(~H)(~N)**
2. **O=C-C-N**
3. O=C-C-C-C-C-N
4. [♯1]-**C-C-N-[♯1]**
5. C(-C)(-C)(=N)
6. **N-C-C-N-C**
7. N=C-C-C
8. >= 2 **any ring size 6**

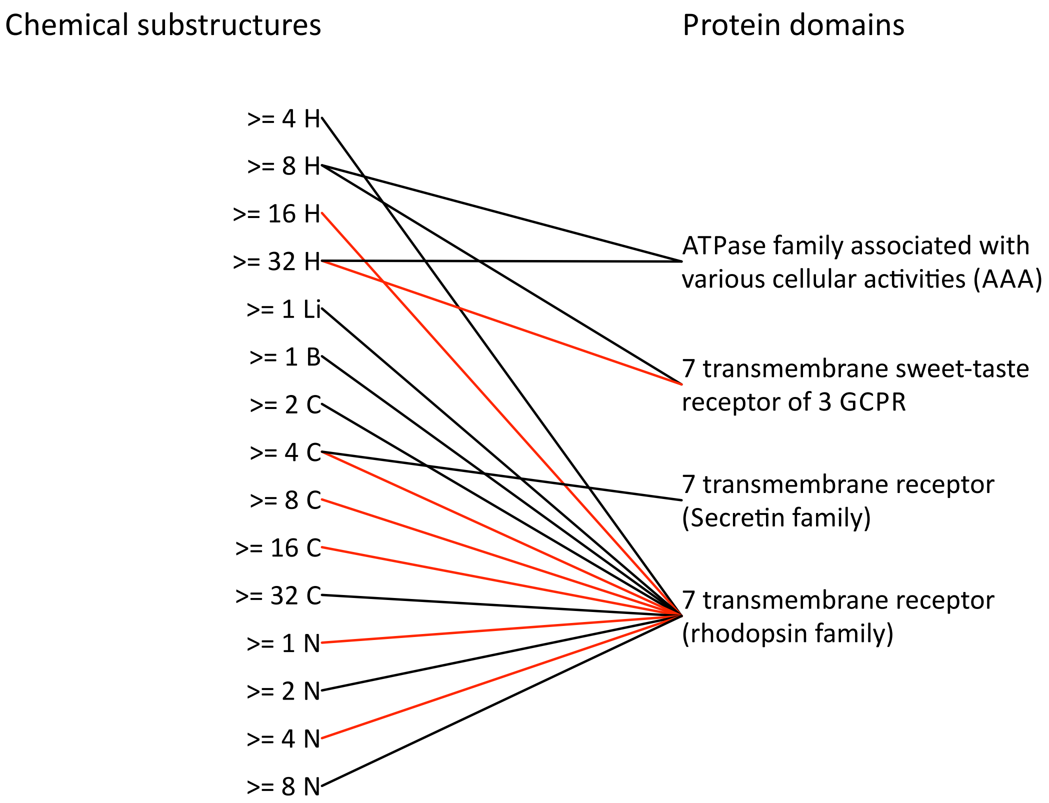

Figure S16: Each cluster is associated with a list of drug features and a list of protein features. Only are kept the features more present in the drugs and proteins of the cluster than in other clusters. The result can be represented as a network that connects chemical substructures and protein domains. The network in this figure was constructed from the 10 clusters in **Figure S13** that have a *p*-value lower than 10^{-7}. The weight of an edge is obtained by computing the product of the importance value associated to the chemical substructure and the protein domain that it connects. In the illustrated network, only are kept the 17 edges with a weight higher than the arbitrary chosen threshold $6 \cdot 10^{-6}$. The 6 red lines represent edges shared by our network and a similar network from [6], corresponding to more the 35% of the 17 edges of the network.

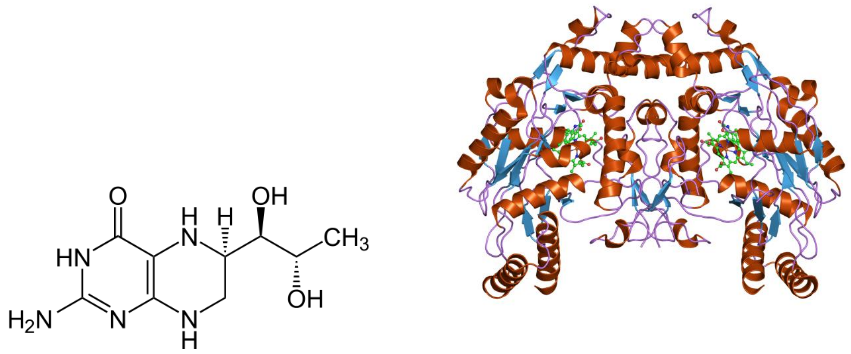

Figure S17: The 5,6,7,8-Tetrahydrobiopterin drug (left) and the NOS3 (endothelial nitric oxide synthase) protein (right) have been proved to interact.

9. C(∼Br)(:N)
10. C(-C)(=N)

Substructures in bold are the ones present in 5,6,7,8-Tetrahydrobiopterin. The seven PFAM domains for which the importance is higher than zero are the following:

1. PDZ domain (also known as DHR or GLGF)
2. **Nitric oxide synthase, oxygenase domain**
3. ACT domain
4. **FAD binding domain**
5. **Oxidoreductase NAD-binding domain**
6. **Flavodoxin**
7. Biopterin-dependent aromatic amino acid hydroxylase

The domains in bold are the ones present in NOS3. These different features are the ones that led the algorithm to give a high probability to the target pair to interact.

References

[1] Kevin Bleakley and Yoshihiro Yamanishi. Supervised prediction of drug-target interactions using bipartite local models. *Bioinformatics*, 25(18):2397–2403, 2009.

[2] L. Breiman, J.H. Friedman, R.A. Olsen, and C.J. Stone. *Classification and Regression Trees*. Wadsworth International, 1984.

[3] Leo Breiman. Random forests. *Machine learning*, 45(1):5–32, 2001.

[4] Feixiong Cheng, Chuang Liu equal contributor, Jing Jiang, Weiqiang Lu, Weihua Li, Guixia Liu, Weixing Zhou, Jin Huang, and Yun Tang. Prediction of drug-target interactions and drug repositioning via network-based inference. *PloS Compuational Biology*, 8(5), 2012.

[5] P. Geurts, D. Ernst, and L. Wehenkel. Extremely randomized trees. *Machine Learning*, 63(1):3–42, 2006.

[6] Y. Yamanishi, E. Pauwels, H. Saigo, and V. Stoven. Extracting sets of chemical substructures and protein domains governing drug-target interactions. *Journal of Chemical Information and Modeling*, page 110505071700060, May 2011.

[7] Yoshihiro Yamanishi, Michihiro Araki, Alex Gutteridge, Wataru Honda, and Minoru Kanehisa. Prediction of drug-target interaction networks from the integration of chemical and genomic spaces. *Bioinformatics*, 24(13):i232–i240, 2008.
